# Diacylglycerol kinase-ε is required for the formation of GPI-anchored CD14 and the LPS-induced proinflammatory responses of macrophages

**DOI:** 10.1186/s12964-026-02884-2

**Published:** 2026-04-25

**Authors:** Aneta Hromada-Judycka, Gabriela Traczyk, Ichrak Ben Amor, Anna Ciesielska, Aniela Mąkosa, Daniel Varon Silva, Katarzyna Kwiatkowska

**Affiliations:** 1 Laboratory of Molecular Membrane Biology, Nencki Institute of Experimental Biology PAS, 3 Pasteur St, Warsaw, 02-093 Poland; 2 Institute for Chemistry and Bioanalytics, School of Life Sciences, FHNW, Hofackerstrasse 30, Muttenz, 4132 Schweiz

**Keywords:** Diacylglycerol kinase, CD14, GPI anchor, LPS, TLR signaling, Pro-inflammatory signaling

## Abstract

**Background:**

Diacylglycerol kinase-ε (DGKε) is a unique member of the DGK family with strict specificity toward SAG, stearic/palmitic and arachidonic fatty acid-containing DAG, which produces phosphatidic acid used for the synthesis of phosphatidylinositol (PI). PI and its derivatives orchestrate numerous processes. These include the pro-inflammatory signaling of Toll-like receptor 4 (TLR4) and its accessory protein, CD14, which are activated in macrophages by bacterial lipopolysaccharide (LPS).

**Methods:**

To assess the role of DGKε in LPS-induced responses, we obtained Raw264.7 cells stably depleted of DGKε and subsequently rescued them with DGKε-Myc. The DGKε-depleted cells were also treated with a synthetic GPI precursor. To assess the activity of DGKε and other DGKs in cellular fractions, a fluorescent assay was used, followed by thin-layer chromatography. RT-qPCR, immunoblotting, ELISA, and flow cytometry were used to examine protein abundance, LPS-induced signaling, and cytokine production. Cytokine expression was also analyzed after TLR2 activation. Cells were fractionated with Triton X-100 and X-114 to examine the distribution of GPI-anchored proteins (GPI-APs). *Dgke* was silenced with siRNA in mouse bone marrow-derived macrophages (BMDM).

**Results:**

SAG phosphorylation was markedly decreased in DGKε-depleted Raw264.7 cells, with the activity of other DGKs unaffected. The DGKε depletion abolished the endosomal TLR4 signaling, engaging TRIF and IRF3. The MyD88-dependent signaling pathway was partially inhibited. No mature, GPI-anchored form of CD14 was produced in the DGKε-depleted cells, and residual amounts of CD14 and other GPI-APs were found on the cell surface. The DGKε depletion also inhibited TLR2-mediated cytokine expression and reduced CD14 level in BMDM. The reintroduction of DGKε in Raw264.7 cells restored SAG phosphorylation, total and cell-surface abundance of GPI-CD14 and other GPI-APs, and TLR4 and TLR2 signaling. Treatment of cells with the synthetic GPI precursor partially restored the cell-surface level of CD14.

**Conclusions:**

DGKε-dependent phosphorylation of SAG controls the biosynthesis of the GPI moiety of CD14, thereby affecting TLR signaling in macrophages. In addition, DGKε can influence the TLR pathways independently of CD14 formation. Together, these findings identify DGKε as a key factor determining the sensitivity of macrophages to LPS and other microbial components.

**Graphical Abstract:**

**Figure:**
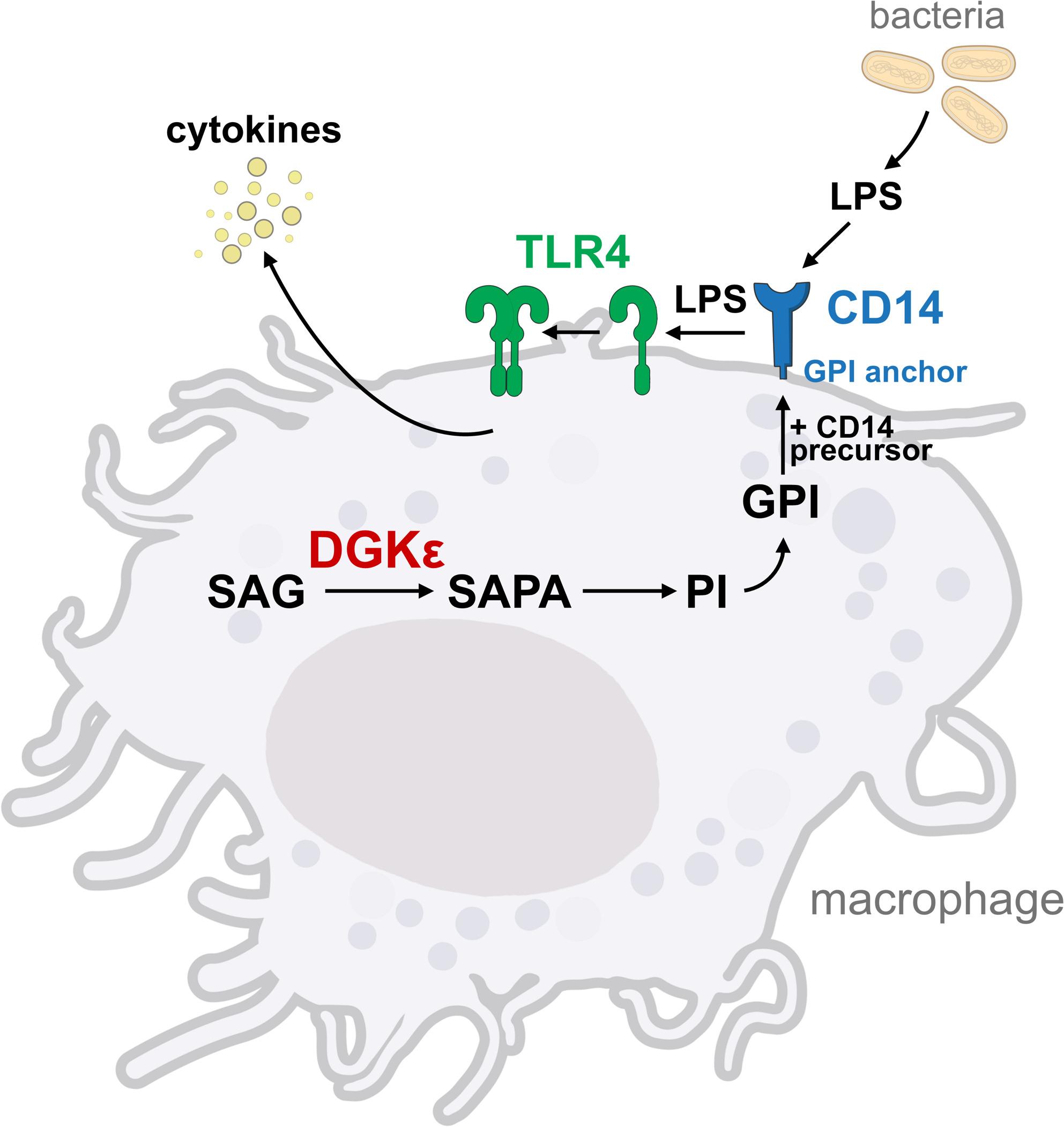
The SAG-to-SAPA phosphorylation by DGKε is required for PI synthesis, which in turn is essential for GPI-CD14 formation in macrophages, affecting pro-inflammatory signaling of TLR4.

**Supplementary Information:**

The online version contains supplementary material available at 10.1186/s12964-026-02884-2.

## Introduction

Lipopolysaccharide (LPS, endotoxin) is a major component of the outer membrane of Gram-negative bacteria belonging to so-called pathogen-associated molecular patterns, PAMPs [1]. Upon infection, LPS and other PAMPs trigger acute pro-inflammatory reactions aiming to eradicate the bacteria. However, when exaggerated and dysregulated, this response can lead to potentially fatal sepsis. Also, prolonged low-grade systemic endotoxemia can occur when dysbiotic microflora increase gut permeability, allowing LPS to enter the bloodstream and paving the way for obesity, diabetes, and cardiovascular disease [2–4]. An important role in the response to LPS is played by macrophages, whose plasma membrane is equipped with the pattern-recognition receptor Toll-like 4 (TLR4) and its accessory protein CD14. In a typical scenario, LPS aggregates released from bacteria are first recognized by serum LPS-binding protein (LBP), which facilitates subsequent binding of LPS monomers to the hydrophobic N-terminal pocket of CD14 [5, 6]. The LPS monomers are then transferred from CD14 onto the complex of TLR4 with covalently linked MD2 [7]. The TLR4/MD2 complexes dimerize and recruit a pair of adaptor proteins, TIRAP and MyD88, and the latter triggers a signaling cascade involving IRAK1/2 kinases and TRAF6 E3 ubiquitin ligase, and also MAP kinases, ultimately leading to the activation of NFκB and AP-1 transcription factors [8–10]. Eventually, pro-inflammatory cytokines are expressed, including the hallmark one, TNFα [11, 12]. Subsequently, CD14 governs the internalization of TLR4/MD2 and in endosomes, TLR4 recruits a second pair of adaptor proteins, TRAM and TRIF, launching a signaling cascade involving TRAF3 E3 ubiquitin ligase and leading to the activation of IRF3/7 transcription factors and production of type I interferons and the chemokine RANTES [13–18]. In addition, a late-phase NFκB activation occurs downstream of the TRIF-TRAF6-dependent pathway [19, 20]. The LPS-induced NFκB activation also allows a subsequent activation and assembly of the NLRP3 inflammasome, which is currently considered a key factor in the development of (auto)inflammatory diseases [21]. It has recently been shown that CD14 delivers LPS to TLR4 residing in intracellular compartments, thereby shaping the endosomal signaling of TLR4 [22].

CD14 is expressed mainly in myeloid cells such as monocytes, macrophages, and dendritic cells and is embedded in the outer leaflet of their plasma membrane via a glycosylphosphatidylinositol (GPI) anchor [23, 24]. CD14 is synthesized as a precursor protein (375 amino acids in human, 366 in mouse) anchored in the endoplasmic reticulum (ER) membrane via its C-terminal transmembrane fragment. The C-terminal 30 amino acids are then cleaved off and replaced by a preassembled GPI moiety in a reaction catalyzed by GPI transamidase; the nascent GPI-linked protein is subjected to GPI anchor remodeling in the ER and transported toward the plasma membrane via the Golgi apparatus. There, the GPI moiety undergoes further remodeling, which drives the protein accumulation in sphingolipid-cholesterol nanodomains (rafts) [25, 26]. Concomitantly, CD14 undergoes *N*- and *O*-glycosylation, resulting in several different forms of mature CD14 [27]. In addition to the plasma-membrane-bound GPI-linked CD14, soluble forms of CD14 are found in the serum, facilitating the activation of even CD14-deficient cells by LPS. The mechanisms governing the generation of soluble CD14 are not well understood, but they are known to include shedding from the cell surface and/or exocytosis, partly in analogy to other GPI-anchored proteins (GPI-APs) [28–33]. The functioning of the LBP-CD14 cascade allows TLR4 to be activated by low (nanomolar) concentrations of the major form of LPS, so-called smooth LPS [34]. However, when present in high concentrations, LPS can also be transferred to TLR4 by albumin, making CD14 dispensable for the activation of the MyD88-dependent pathway [35, 36]. In contrast, the participation of membrane-anchored CD14 is required for the endocytosis of TLR4/MD2 and/or for delivering LPS to intracellular TLR4, thereby initiating the endosomal TRIF-dependent TLR4 signaling pathway in macrophages [14, 18, 22], which underscores the signaling functions of GPI-anchored CD14. Consistent with this, our studies have demonstrated that upon LPS binding, CD14 induces a biphasic generation of phosphatidylinositol 4,5-bisphosphate (PI(4,5)P_2_) in rafts [37]. A line of studies have revealed some details on how PI(4,5)P_2_ interacts with rafts and how it and products of its hydrolysis and phosphorylation control the LPS-induced signaling [38–43].

PI(4,5)P_2_ and other derivatives of PI are important regulatory lipids controling, among others, signal transduction (including the LPS-triggered one), vesicular trafficking, non-vesicular lipid transport, and cytoskeleton organization, which makes them of substantial interest in diverse research fields [44, 45]. The parental molecule, PI, is also a versatile lipid used for GPI synthesis and as a source of arachidonic acid for eicosanoid production [46, 47]. PI is synthesized *de novo* in the ER through a multistep process from glycerol-3-phosphate. This synthesis is likely combined with fatty acid remodeling in the Lands cycle to achieve the stearic and arachidonic fatty acid composition (C38:4) typical of PI [48–52]. Additionally, PI can be generated in the so-called PI cycle taking place at the ER and plasma membrane, which can intertwine with the *de novo* PI synthesis and serves to rebuild the PI level after stimulus-induced hydrolysis of PI(4,5)P_2_ into diacylglycerol (DAG) and inositol 1,4,5-trisphosphate (IP_3_) [48, 49]. Despite the evident importance of PI and its derivatives for LPS-induced responses, the molecular mechanisms controling their abundance and turnover, as well as their relation to CD14 remain poorly understood. Studying those questions, we have found that several enzymes involved in the PI cycle are palmitoylated in an LPS-dependent manner [53]. These include diacylglycerol kinase-ε (DGKε), whose palmitoylation was previously unknown [53]. DGKε is a member of a family of ten enzymes that phosphorylate DAG to phosphatidic acid (PA), thereby controling cellular levels of these two key intermediates in lipid synthesis, with some of their species also functioning as signaling molecules [54]. Among all the DGKs, only DGKε is an integral membrane protein anchored in the membrane via an N-terminal α-helix. We found that DGKε is *S*-palmitoylated at Cys 38/40 (mouse/human) of this α-helix [55]. Importantly, only DGKε displays strict specificity toward DAG bearing the C18:0 (stearic)/C16:0 (palmitic) and C20:4 (arachidonic) fatty acids at the *sn*-1 and *sn*-2 position, respectively, and therefore abbreviated SAG. Its phosphorylation yields to 1-stearoyl-2-arachidonoyl-phosphatidic acid (SAPA) [56–59]. As the predominant form of PI bears exactly the same C38:4 fatty acids, it can be derived in the PI cycle from SAPA, which in turn is synthesized from SAG, suggesting a critical role of DGKε in an unhindered operation of the PI cycle [60]. However, a contribution of DGKs other than DGKε to the PI cycle has recently been implicated, with a concomitant suggestion that the cycle also provides PI in resting cells [50, 61].

To examine the contribution of DGKε to LPS-induced signaling, we obtained Raw264.7 cells depleted of DGKε by shRNA silencing and subsequently rescued them with Myc-tagged DGKε. Unexpectedly, we found that DGKε is indispensable for the formation of mature, GPI-anchored CD14 and other GPI-APs in macrophages, likely by regulating the GPI anchor synthesis. DGKε activity may also have additional effects, ultimately influencing signaling of TLR4 and TLR2. We thereby identified DGKε as a key factor affecting the response of macrophages to LPS and other PAMPs.

## Methods

### Cell culture and stimulation

Raw264.7 cells (ATCC) were cultured in DMEM containing 10% FBS (Thermo Fisher Scientific) and 4.5 g/l glucose. Primary bone marrow-derived macrophages (BMDM) were obtained from C57BL/6 mice and confirmed by staining with anti-F4/80 according to Toda et al., [62]. To silence *Dgke*, 2 × 10^6^ BMDM were transfected with 100 pmol of DGKε siRNA or negative control siRNA (Qiagen, cat. Nos. SI00978439 and 1022076, respectively) using Mouse Macrophage Nucleofector Kit (Lonza, cat No. VPA-1009) and Amaxa Nucleofector II Device (Lonza) according to the manufacturer’s instructions, and used for experiments after 24 h. Before experiments, the medium was replaced with a fresh one for 2 h. Cells were left unstimulated or were stimulated with 10 or 100 ng/ml smooth LPS from *Escherichia coli* O111:B4 (List Biological Laboratories) at 5% CO_2_, 37 °C for up to 4 h. In a series of studies, cells were stimulated with 10 ng/ml *N-*palmitoyl-*S*-[2,3-bis(palmitoyloxy)-propyl]-(*R*)-cysteinyl-seryl-(lysyl)3-lysine (Pam_3_CSK_4_) or 10 ng/ml *S*-[2,3-bis(palmitoyloxy)-propyl]-(*R*)-cysteinyl-seryl-(lysyl)3-lysine (Pam_2_CSK_4_) (both from InvivoGen).

### Construction of Raw264.7 cells with silenced and rescued expression of *Dgke*

To obtain Raw264.7 cells depleted of DGKε, the cells were transfected with lentiviral particles containing five different *Dgke*-targeting shRNA or non-mammalian shRNA for control (Merck, Supplementary Table 1). To reintroduce DGKε, DGKε KD cells were transfected with lentiviral particles bearing the DGKε sequence with a double Myc tag added at the C-terminus (custom-made by OriGene). The efficiency of *Dgke* silencing and its reversion was verified with RT-qPCR using primers specific to the *Dgke* gene, with *Tbp* as a reference (Supplementary Table 2, see also [63]). Further details on cell preparation are provided in the Supplementary Methods.

### Treatment of cells with synthetic compound 1 

Compound 1, 2-acetamido-2-deoxy-α-D-glucopyranosyl-(1→6)-1-*O*-(2-oleoyl-1-stearoyl-*sn*-glycero-3-phosphonate)-D-*myo*-inositol, was synthesized, dissolved in DMSO at 10 mM concentration, and used as previously described [64]. Briefly, cells were seeded and cultured for 24 h, and then switched to serum-free DMEM containing either 50 µM compound 1 or 0.5% DMSO for 18 h. Subsequently, the medium was replaced for 2 h with DMEM supplemented with 10% FBS. The cells were then either lysed in a buffer containing 0.5% TX-100, 100 mM NaCl, 2 mM EDTA, 2 mM EGTA, 30 mM Hepes, pH 7.4, protease inhibitors (1 mM PMSF, 2 µg/ml aprotinin, 2 µg/ml leupeptin, 0.7 µg/ml pepstatin), phosphatase inhibitors (10 mM *p*-nitrophenyl phosphate, 1 mM Na_3_VO_4_, 50 µM phenylarsine oxide), and 250 U/ml Benzonase Nuclease (Merck) for whole cell lysate analysis, or subjected to fractionation in TX-100 or to flow cytometry, as described below.

### Fractionation of TX-100 lysates of cells

Cells (1 × 10^6^ per sample) were fractionated into TX-100-soluble, TX-100-insoluble (also called detergent-resistant DRM), and SDS-soluble fractions essentially as described earlier, with the DRM fraction corresponding roughly to membrane rafts [65]. Equivalent volumes of the fractions were subjected to SDS-PAGE followed by immunoblotting.

### Flow cytometry

Cell-surface CD14, TLR4, and uPAR were analyzed by flow cytometry using antibodies listed in Supplementary Table 3 as described by Matveichuk et al., [65]. Total GPI-APs were stained with FLAER (Spark Blue 488 FLAER, BioLegend, cat. No. 567904) in 1% FBS for 30 min, according to the manufacturer’s instructions. In a series of experiments, cells were treated with 0.2 U/ml phosphatidylinositol-specific phospholipase C (PI-PLC; Invitrogen, cat. No. P6466) for 1 h at 37 °C [37] before the labeling. Cell fluorescence was determined with a BD FACS Calibur or BD LSR Fortessa flow cytometers. FITC/Spark Blue 488, phycoerythrin, and Alexa Fluor 647 fluorescence were detected using 530/30 nm, 585/42 nm, and 670/14 nm band pass, respectively. Data were analyzed using BD CellQuest Pro or FACSDiva software (BD Biosciences), and the amounts of cell-surface CD14, TLR4, uPAR, and total GPI-APs were calculated based on the geometric mean of fluorescence intensity, as described earlier [65].

### Extraction of GPI-anchored proteins with TX-114

To analyze the partition of membrane proteins with TX-114, the detergent (Thermo Fisher Scientific cat. No. 422360025) was precondensed to 12% according to Taguchi and Schatzl [66]. Cells (2 × 10^6^ per sample) were homogenized by sonication in 200 µl of the homogenization buffer (5 mM EDTA, 1 mM PMSF, 20 µg/ml aprotinin, 20 µg/ml leupeptin, 20 mM Tris, pH 7.4), supplemented with 250 mM sucrose, and subjected to centrifugation (1,000 x *g*, 10 min, 4 °C) to obtain the post-nuclear supernatant (PNS). Next, the PNS was ultracentrifuged (200,000 x *g*, 1 h, 4 °C), and the obtained supernatant containing cytosolic proteins was collected. The pellet (membrane fraction) was dissolved in 200 µl of the PI-PLC reaction buffer containing 20 mM Tris, 0.1% TX-100, protease inhibitors as above, pH 7.5 [67]. The lysate was divided in half and supplemented or not with 2 U/ml PI-PLC. After 1 h (37 °C), TX-114 was added to both samples to 2% final concentration. After a 10-min incubation on ice, the samples were centrifuged (1,000 x *g*, 10 min, 37 °C) and separated into aqueous (upper) and detergent (lower) phase, the latter washed once by centrifugation with PI-PLC reaction buffer (37 °C); both samples were supplemented with respective buffer to keep sample conditions and volumes consistent [67]. Proteins were precipitated from each fraction with methanol: chloroform: water (3:1:4, v:v:v), dissolved in SDS-sample buffer, and equivalent volumes were subjected to SDS-PAGE.

### DGKε activity assay

DGKε activity was determined using the mixed micelle activity assay with 1-NBD-stearoyl-2-arachidonoyl-*sn*-glycerol (NBD-SAG, Cayman Chemical, cat. No. 10011300) developed and characterized by us earlier [55, 63]. Further details on cell lysis and preparation of micelles are provided in the Supplementary Methods. The enzymatic reaction was carried out for 10 min (24 °C), then lipids were extracted and separated by thin-layer chromatography (TLC) on silica gel 60 (Merck) (1/5 − 1/10 of the reaction mixture) along with a standard, 0.5–50 pmol of 1-palmitoyl-2-NBD-dodecanoyl-*sn*-glycero-3-phosphate (NBD-PDPA, Avanti, cat. No. 810174), with chloroform: methanol: acetic acid 80% (65:15:5, v:v:v) as a mobile phase. NBD-lipids, including 1-NBD-stearoyl-2-arachidonoyl-*sn*-glycero-3-phosphate (NBD-SAPA) and NBD-PDPA were visualized using G: Box (Syngene), and fluorescence intensity was assessed with ImageJ software using a standard curve drawn for NBD-PDPA and corrected by background subtraction.

In a series of experiments, DGKε activity and the activity of other DGKs were determined in cell fractions. For this purpose, cells (3 × 10^6^ per sample) were homogenized (300 µl of 1 mM EDTA, 1 mM EGTA, 1 mM DTT, 1 mM PMSF, 20 µg/ml aprotinin, 20 µg/ml leupeptin, 20 mM Tris-HCl, pH 7.4), supplemented with 250 mM sucrose, centrifuged to obtain PNS, which was then supplemented with 1 M NaCl and ultracentrifuged (200,000 x *g*, 1 h, 4 °C). The obtained supernatant containing cytosolic proteins was collected, pellet (membrane fraction) was dissolved in homogenization buffer supplemented with 1 M NaCl, and protein concentration was determined in all fractions. Subsequently, 40 µg of total protein was used for the DGKε activity assay and analyzed as for cell lysates. In another set of experiments, the treatment of samples with 1 M NaCl was omitted. Alternatively, to analyze the activity of other DGKs, mixed micelles containing 1-NBD-decanoyl-2-decanoyl-*sn*-glycerol/1,2-didecanoyl-*sn*-glycerol (NBD-DDG/DDG at a 1:9 molar ratio, both from Cayman Chemical, cat. Nos. 9000341 and 62210) were used and the reaction buffer was supplemented with 10 mM CaCl_2_ instead of 1 mM EGTA; the 1-NBD-decanoyl-2-decanoyl-*sn*-glycero-3-phosphate (NBD-DDPA) produced was analyzed as above.

In parallel, cell lysates and fractions were supplemented with 2% SDS, vortexed and incubated at room temperature for 15 min, supplemented with SDS-sample buffer, incubated again for 15 min, heated for 10 min at 95 °C and subjected to SDS-PAGE and immunoblotting (antibodies listed in Supplementary Table 3).

### RNA isolation and RT-qPCR

RNA was isolated from cells using the Universal RNA purification kit (EURx) and reverse-transcribed into cDNA using the High-Capacity cDNA Reverse Transcription Kit (Thermo Fisher Scientific) according to the manufacturer’s instructions. For cDNA synthesis, 50 ng of RNA was used in a 10 µl reaction mixture. This amount of input RNA was within the linear range of standard curves generated for determining the efficiency of all primers used. qPCR was performed in a StepOnePlus or QuantStudio5 instruments using Fast SYBR Green Master Mix (Thermo Fisher Scientific). The primers and PCR conditions were described earlier [63, 65] or are shown in Supplementary Table 2. mRNA levels for the investigated genes were calculated relative to the mRNA level for the *Tbp* or *Hprt* gene, each variant run in duplicate. Either the ΔCt (relative expression) or the ΔΔCt method (fold change) was applied, as specified in the figure legends. The ΔΔCt method was applied according to Livak and Schmittgen [68].

### Cytokine assays

TNFα and CCL5/RANTES were quantified in cell culture supernatants using mouse ELISA kits (BioLegend, R&D Systems). The profiles of all secreted inflammatory markers were assayed using the Mouse Cytokine Array Kit, Panel A (R&D System), as described earlier [65, 69].

### Immunoblotting

Proteins were separated by 10% SDS-PAGE and transferred to nitrocellulose membranes which were subsequently probed with antibodies listed in Supplementary Table 3. Precision Plus Protein Dual Color Standards (Bio-Rad, cat. No. 161–0374) and PageRuler Prestained Protein Ladder (Thermo Fisher Scientific, cat. No. 26616) were used as molecular weight standards. Immunoreactive bands were detected by chemiluminescence and analyzed densitometrically using ImageJ software, as described earlier [53, 65, 70].

### Data analysis

The significance of differences was assessed using one-way ANOVA or two-way ANOVA with interaction, followed by Tukey’s multiple comparison post hoc. For analysis of data sets not meeting the Brown-Forsythe test requirements for homogeneity of variances, Welch’s ANOVA and Dunnett’s T3 post hoc were applied. Calculations were performed using GraphPad Prism software (version 10.6.1). Differences were considered statistically significant if *p* < 0.05. The statistical test used for analysis is specified in the figure legends. For clarity, not all of the significant differences are marked in the figures. Bar graphs were prepared using Microsoft Excel. Each data point shown as a dot on the histograms represents one biological replicate.

## Results

### Characteristics of macrophages depleted of DGKε and rescued with DGKε-Myc

**Fig. 1:**
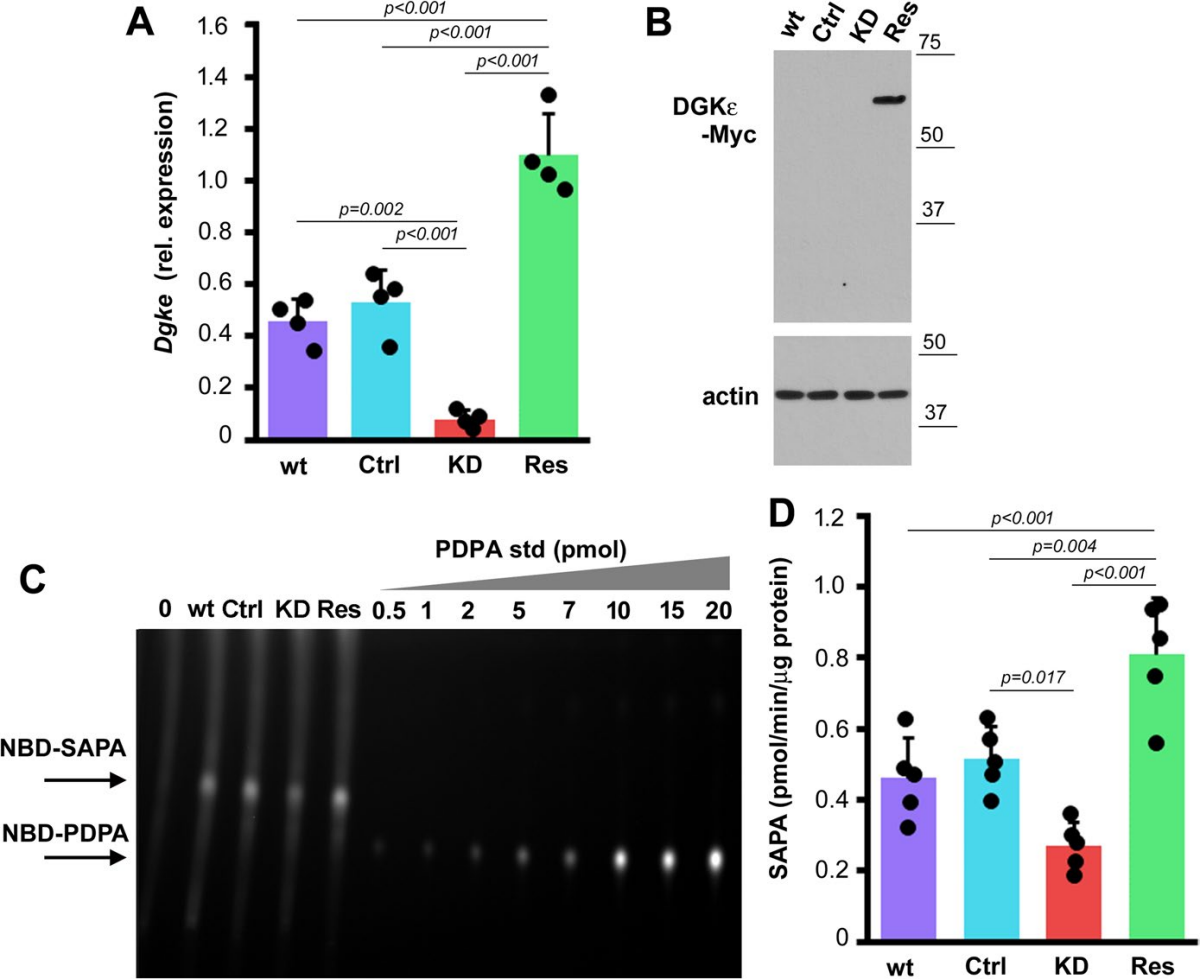
Knockdown of *Dgke* and its rescue with DGKε-Myc in Raw264.7 cells. **A** RT-qPCR analysis of the relative DGKε mRNA level in the obtained variants of Raw264.7 cells (wt). Transcripts were quantified by RT-qPCR relative to *Tbp.* **B** Production of DGKε-Myc in DGKε-Myc-rescued cells revealed by immunoblotting with anti-Myc antibody in cell lysates. Positions of molecular weight markers are shown on the right in kDa. **C**, **D** Phosphorylation of SAG to SAPA quantified in cell lysates by the fluorescence assay. **C** Representative TLC separation revealing NBD-SAPA production. The reaction mixture contained 50 µg of total protein; lipids from 1/5 of the reaction mixture were applied onto the plate. NBD-labeled lipids were separated by TLC together with 0.5–20 pmol NBD-PDPA used to draw a standard curve for each experiment. “0” – no lysate added. **D** SAPA production based on densitometric analysis of NBD-SAPA and the calibration curve for NBD-PDPA. Data shown in (**A**, **D**) are mean ± SD from four (**A**) or five (**D**) biological replicates. Each point represents one biological replicate. Significantly different values, as indicated by one-way ANOVA with Tukey’s post hoc test, are marked

To reveal the role of DGKε in LPS-induced signaling, we obtained Raw264.7 cells with stably silenced expression of *Dgke* using shRNA in lentiviral particles, followed by puromycin selection. Among the five shRNA species used, variants No. 1 and No. 5 were the most effective in reducing the relative DGKε mRNA level in both resting and LPS-stimulated cells. Control non-mammalian shRNA did not interfere with the DGKε mRNA level (Supplementary Fig. 1). Subsequently, we reintroduced DGKε Myc-tagged at the C terminus to the Raw264.7 transfectants depleted of DGKε with shRNA variant No. 1, and selected the rescued cells by G418 resistance. In parallel, the respective control and DGKε-depleted cells were subjected to the second round of transfection with an empty vector carrying only the G418-resistance gene. In this manner, we obtained: control cells, in which the relative DGKε expression was unaffected compared to parental Raw264.7 cells, DGKε knockdown cells with the relative DGKε mRNA level reduced by about 85%, and cells expressing DGKε-Myc at a level approximately 2.1-fold higher than endogenous DGKε, called the DGKε-Myc-rescued variant (Fig. 1A). We confirmed the production of DGKε-Myc protein in the rescued cells (Fig. 1B). The down-regulation of DGKε in DGKε-KD cells reduced the phosphorylation of SAG to SAPA in cell lysates by about 41–48% (vs. wt and Ctrl cells). It was fully restored in DGKε-Myc-rescued cells, surpassing the activity in control cells 1.6-1.8-fold (Fig. 1C-D).

**Fig. 2:**
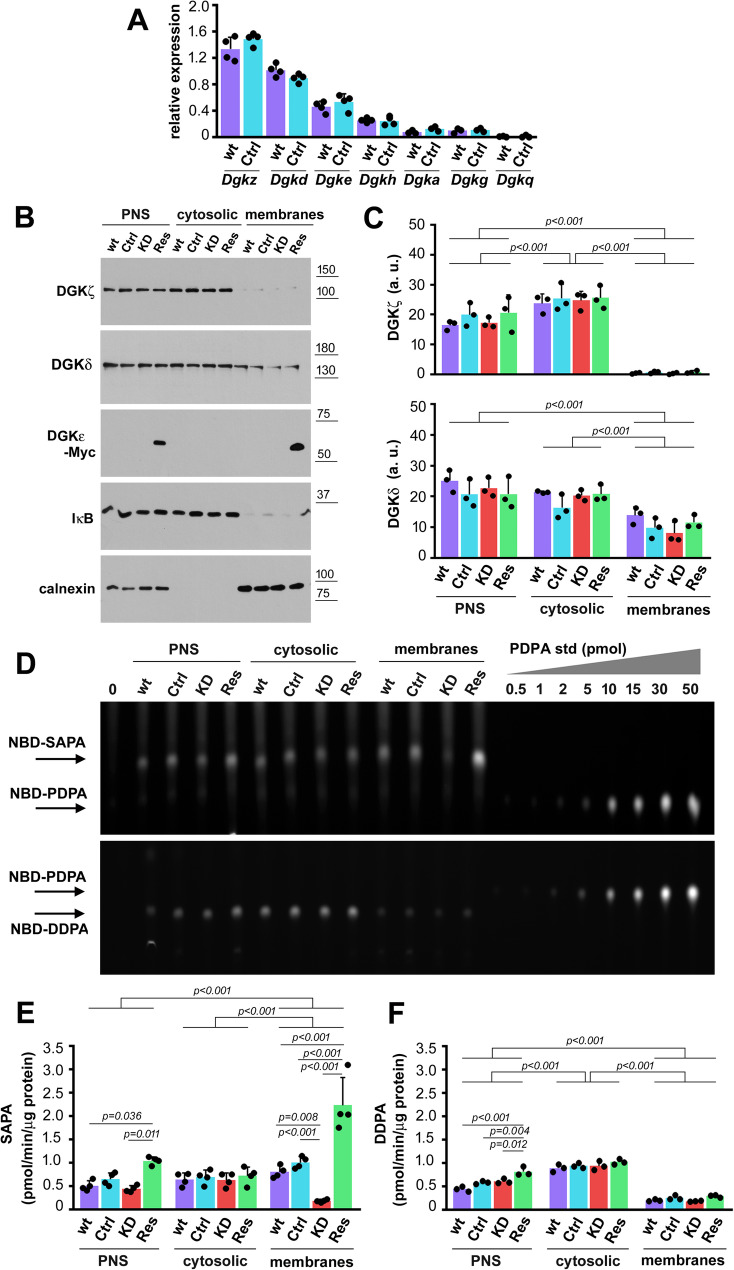
SAG phosphorylation is affected by DGKε knockdown and rescue. **A** Relative expression of *Dgkz*,* d*,* e*,* h*,* a*,* g*,* and q* in Raw264.7 (wt) and Ctrl cells. Transcripts were quantified by RT-qPCR relative to *Tbp.* **B**-**F** PNS obtained from wt, Ctrl, DGKε-KD, and DGKε-Myc-rescued cells homogenates were supplemented with 1 M NaCl and fractionated into cytosolic and membrane fractions. **B** Distribution of indicated proteins in the fractions determined by immunoblotting. Equal amounts of protein, 10 µg, were loaded in each lane of the gel. Positions of molecular weight markers are shown on the right in kDa. **C** Abundance of DGKζ (upper panel) and DGKδ (lower panel) in each fraction determined by densitometry. Data shown are mean ± SD. **D**-**F** Phosphorylation of SAG to SAPA (upper panel in **D** and **E**) and DDG to DDPA (lower panel in **D** and **F**) in cell fractions determined using the fluorescence assay. **D** Representative TLC results revealing NBD-SAPA (upper panel) and NBD-DDPA (lower panel) production. The reaction mixture contained 40 µg of total protein; lipids from 1/5 of the reaction mixture were applied onto the plate. NBD-labeled lipids were separated by TLC together with 0.5–50 pmol NBD-PDPA used to draw a standard curve for each experiment. “0” - no homogenate added. **E**, **F** Production of (**E**) SAPA and (**F**) DDPA based on densitometric analysis of NBD-SAPA or NBD-DDPA and a calibration curve for NBD-PDPA. Data shown are mean ± SD from three (**C**, **F**) or four (**A**,** E**) biological replicates. Each point represents one biological replicate. Because equal amounts of protein were loaded on the gels and used for SAG phosphorylation, proteins in both the cytosolic and membrane fractions are enriched compared to the PNS. In (**C**, **E**, **F**), significantly different values, as indicated by two-way ANOVA with Tukey’s post hoc test, are marked

The SAG-specific activity remaining in DGKε-KD cell lysates could be ascribed to residual DGKε and/or DGKs other than DGKε, whose accessibility to SAG was facilitated by the cell lysis. Among the seven *Dgk* genes likely to be expressed in monocytes/macrophages [71], *Dgkz*, *Dgkd*, and *Dgkh* (encoding DGKη) were found to be expressed relatively abundantly in Raw264.7 and Ctrl cells, exceeding or being comparable to the level of *Dgke*, while *Dgka*, *Dgkg*, and *Dgkq* (encoding DGKθ) showed low relative expression (Fig. 2A, Supplementary Fig. 2). No expression of *Dgkk* was found, as could be expected; *Dgkb* and *Dgki* are specifically expressed in neuronal cells [71, 72]. It should be emphasized that the above analysis provides only a rough estimate of the mRNA level proportions of various DGKs and that an absolute quantification of the exact gene copy number would require further studies [73]. Nevertheless, the relative expression of *Dgka* and *Dgkg* was found to be reduced in DGKε-KD cells, returning to the control level in DGKε-Myc-rescued cells (Supplementary Fig. 2), suggesting a DGKε-dependent regulation. *Dgka* and *Dgkg* mRNA levels are regulated at multiple levels, including FoxO-dependent transcriptional control of *Dgka* [74]; however, the regulation of these processes in macrophages is currently unclear.

To address the issue of the possible contribution of DGKs other than DGKε to the SAG phosphorylation, we fractionated homogenates of the studied cells into cytosolic and membrane fractions in the presence of 1 M NaCl to extract DGKs possibly bound to the surface of membranes [75, 76]. Across all cell types tested, we found comparable levels of DGKζ and DGKδ in the output PNS, indicating that changes in *Dgkz* or *Dgkd* transcript levels did not affect the protein abundance (Fig. 2B, C, see also Supplementary Fig. 3A, B). After fractionation of NaCl-treated PNS, virtually all DGKζ and most of DGKδ was found in the cytosolic fraction marked by the presence of IκB (Fig. 2B, C), while DGKε was located exclusively in the membrane fraction, as marked by the distribution of DGKε-Myc and calnexin, a transmembrane protein of the ER (Fig. 2B). Notably, the activity of the cytosolic DGKs toward SAG was not changed in DGKε-KD or DGKε-Myc-rescued cells compared with controls (Fig. 2D, upper panel; and Fig. 2E). In contrast, the SAG phosphorylation in the NaCl-stripped membrane fraction was lower by about 80% in DGKε-KD cells vs. controls and in DGKε-Myc-rescued cells its level was about 2.5-fold higher than in the controls, closely reflecting the levels of DGKε mRNA in these cells (Fig. 2D, upper panel; and Fig. 2E, compare with Fig. 1A). Taken together, the data indicate that the SAG phosphorylation efficiency correlated with the level of DGKε in the cells. In comparison, when PNS samples were fractionated without the addition of 1 M NaCl, the entire pool of DGKζ and the vast majority of DGKδ remained associated with membranes (Supplementary Fig. 3A, B). Under these conditions, the SAG phosphorylation detected in the cytosol was markedly lower than after NaCl treatment, whereas that in the membrane fraction remained high, except for DGKε-KD cells (Supplementary Fig. 3C and 3D, compare with Fig. 2D upper panel, and Fig. 2E). This indicated the effectiveness of 1 M NaCl in dissociating the DGKs other than DGKε from the membrane surface and their shift to the cytosolic fraction.

In agreement, in the NaCl-stripped membranes, only a low level of phosphorylation of another DAG species, 1,2-didecanoyl-*sn*-glycerol (DDG), was detected, in line with the specificity of DGKε toward SAG. In contrast, DDG was efficiently phosphorylated by the cytosolic fraction harboring DGKs other than DGKε. This phosphorylation was not affected in DGKε-KD or DGKε-Myc-rescued cells. In the input PNS samples, DDG phosphorylation was elevated in DGKε-Myc-rescued cells compared to all other cell types, potentially indicating a DGKε-dependent upregulation of the activity of other DGK(s) in vitro. The significance of this observation has not been investigated further (Fig. 2D, lower panel, and Fig. 2F). Taken together, the observed differences in the rate of SAG-to-SAPA conversion between the cell variants tested reflected differences in their DGKε levels. At the same time, the activity of other DGKs remained largely unaffected. These findings suggest that the cellular level of SAPA is controled primarily, if not exclusively, by DGKε.

### Depletion of DGKε inhibits cytokine production in LPS-stimulated cells

**Fig. 3:**
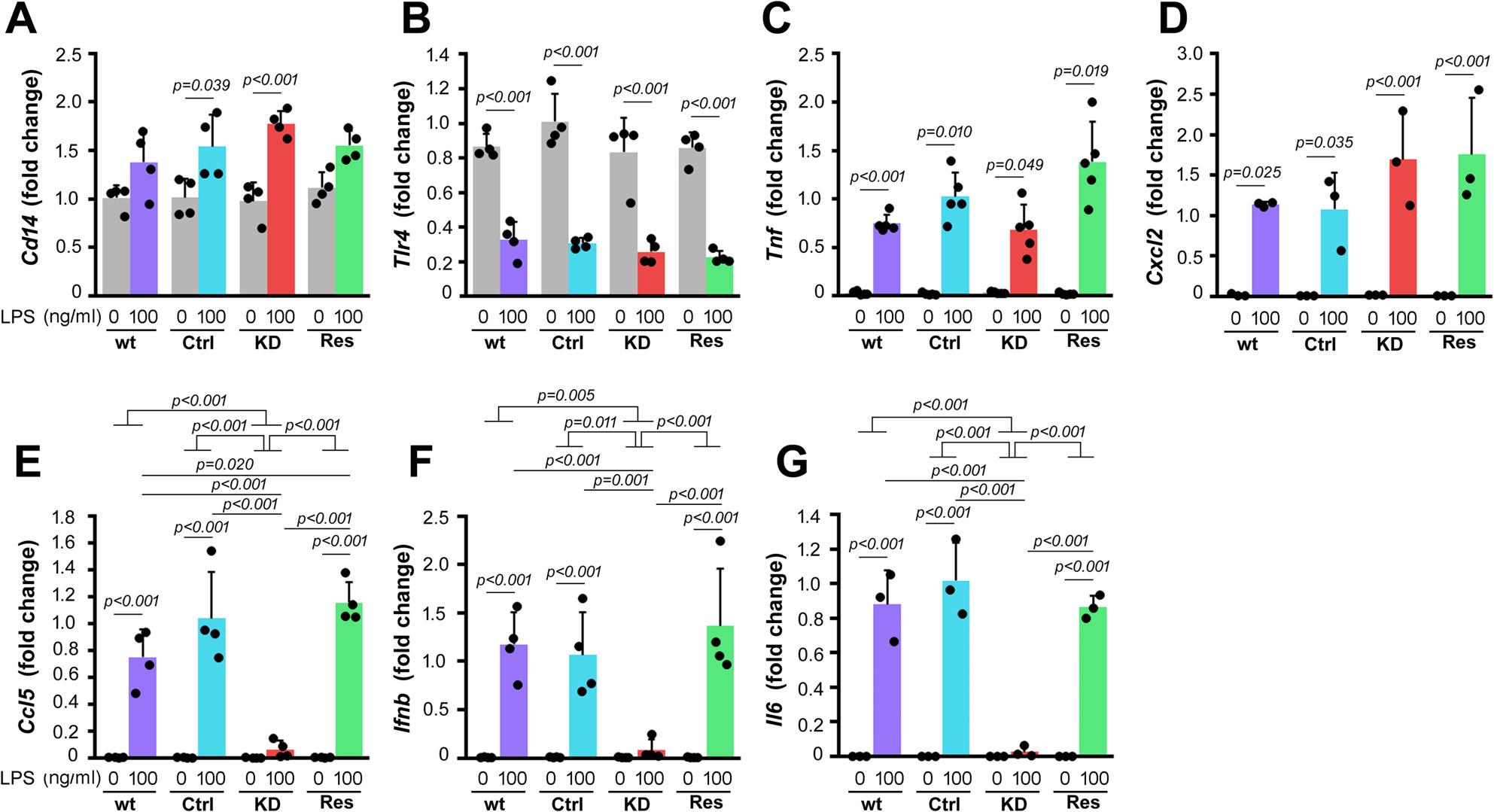
LPS-induced expression of cytokines is down-regulated to varying extents by DGKε knockdown and is rescued by DGKε-Myc. Cells were left unstimulated or were stimulated with 100 ng/ml LPS (4 h). **A**-**G** Transcripts of *Cd14* (**A**), *Tlr4* (**B**), *Tnf* (**C**), *Cxcl2* encoding MIP-2 (**D**), *Ccl5* encoding RANTES (**E**), *Ifnb* (**F**), and *Il6* (**G**) were quantified by RT-qPCR relative to *Hprt* (**A**, **C**, **E**) or *Tbp* (**B**, **D**, **F**, **G**). Unstimulated (**A**, **B**) or stimulated (**C**-**G**) Ctrl was set to 1. Data shown are mean ± SD from three (**D**, **G**), four (**A**, **B**, **E**, **F**), or five (**C**) biological replicates. Each point represents one biological replicate. In (**E**-**G**) and (**C**) significantly different values, as indicated by two-way ANOVA with Tukey’s post hoc test and Welch’s ANOVA with Dunnett’s T3 post hoc test, respectively, are marked. In (**A**, **B**, **D**) the two-way ANOVA indicated no significant differences between cell variants; therefore, differences between unstimulated and stimulated cells were analyzed using one-way ANOVA with Tukey’s post hoc test

We next examined whether DGKε depletion and reintroduction affected LPS-induced responses, starting with the analysis of *Tlr4* and *Cd14* expression. TLR4 and CD14 relative mRNA levels did not differ among DGKε-KD, DGKε-Myc-rescued, control, and parental Raw264.7 cells, both before and after stimulation with LPS (100 ng/ml, 4 h). LPS induced an increase of CD14 mRNA abundance (1.5-1.8-fold, the highest in DGKε-KD cells) and a decrease of TLR4 mRNA (more than 2-fold) in all the cells (Fig. 3A, B).

Despite the unaffected expression of *Tlr4* and *Cd14*, the LPS-stimulated expression of cytokines was defective in cells depleted of DGKε, although the extend of the defect varied depending on the TLR4 signaling pathway involved. Thus, the relative mRNA level of TNFα, induced mainly in the MyD88-dependent manner, tended to be reduced in comparison with Ctrl cells (Fig. 3C). The relative mRNA level of MIP-2, another cytokine produced in the MyD88-dependent pathway, was not affected (Fig. 3D). In contrast, the expression of genes encoding RANTES and IFN-β, cytokines strictly dependent on the endosomal TLR4 signaling, and also IL-6, whose mRNA stability is TRIF-dependent in LPS-stimulated macrophages [11, 16, 17, 77, 78], were virtually abolished in the DGKε-KD cells. Their expression was fully restored in DGKε-Myc-rescued cells (Fig. 3E-G). Overall, these results indicate that DGKε is required for the efficient response of macrophages to LPS. Additionally, we confirmed that the obtained Ctrl and DGKε-Myc-rescued cells reasonably resembled the parental Raw264.7 cells in their LPS-induced production of pro-inflammatory cytokines, with some increased potency observed in the rescued cells.

**Fig. 4:**
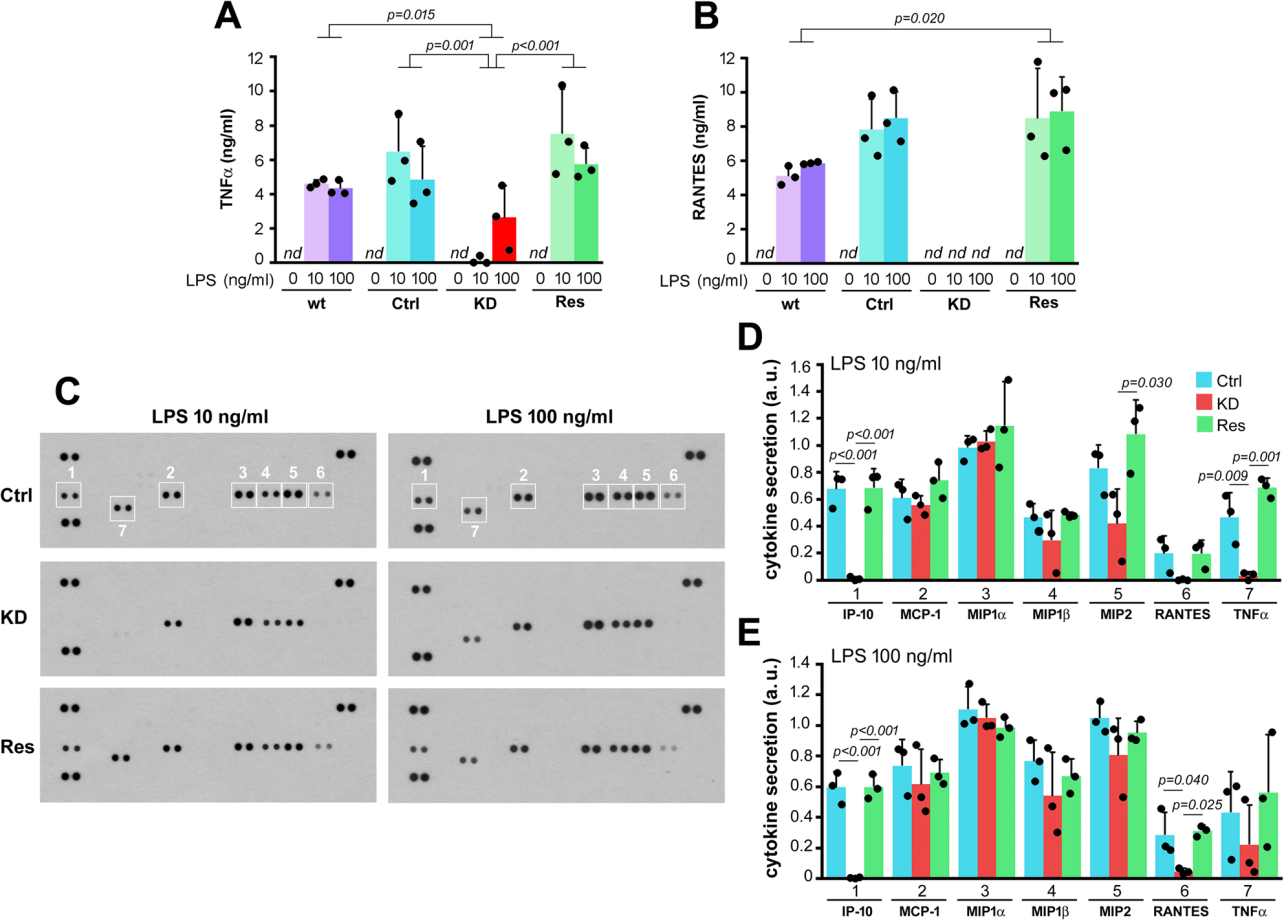
LPS-induced production of cytokines is dependent to varying extents on DGKε. Cells were left unstimulated or were stimulated with 10 ng/ml or 100 ng/ml LPS (4 h). **A**, **B** The concentration of TNFα (**A**) and CCL5/RANTES (**B**) in culture supernatants determined by ELISA. **C**-**E** Cytokine production in cells stimulated with 10 ng/ml or 100 ng/ml LPS determined using a cytokine array (**C**) and quantified by densitometric analysis of the array (**D**, **E**). Dots left unmarked in the upper panels of (**C**) served as internal standards used to normalize signals between membranes. Data shown are mean ± SD from three biological replicates. Each point represents one biological replicate. Significantly different values, as indicated by two-way ANOVA (**A**, **B**) and one-way ANOVA (**D**, **E**), both with Tukey’s post hoc test, are marked. *nd*, not detected; excluded from statistical analysis

We pursued the studies by determining the secretion of TNFα and RANTES in cells stimulated with 10 and 100 ng/ml LPS for 4 h. The depletion of DGKε strongly inhibited the TNFα production induced by 10 ng/ml LPS and abrogated the RANTES production at both LPS concentrations (Fig. 4A, B). Notably, at 100 ng/ml LPS, the TNFα production tended to be lower in DGKε-KD cells vs. Raw264.7 and Ctrl cells (Fig. 4A), in agreement with the TNFα mRNA assessments (Fig. 3C). Importantly, the reintroduction of DGKε restored the TNFα and RANTES production stimulated with both 10 or 100 ng/ml LPS (Fig. 4A, B).

These observations were confirmed by an analysis of the production of a whole array of inflammatory markers in cells stimulated with 10 or 100 ng/ml LPS. We found a virtual abrogation of the secretion of IP-10 and RANTES (both triggered by endosomal TLR4) in DGKε-KD cells and their complete restoration in the DGKε-Myc-rescued cells (Fig. 4C-E). In contrast, the production of the mainly MyD88-dependent MIP-1β, MIP-2, and TNFα tended to be inhibited, reaching statistical significance for MIP-2 and TNFα at 10 ng/ml LPS only (Fig. 4C-E). Taken together, the results indicated that the DGKε depletion inhibits LPS-induced production of pro-inflammatory cytokines, particularly those dependent on TLR4 endocytosis and TRIF involvement, and, to some extent, also the MyD88-dependent ones, especially at the lower LPS concentration.

### Depletion of DGKε inhibits CD14-dependent signaling of TLR4

**Fig. 5:**
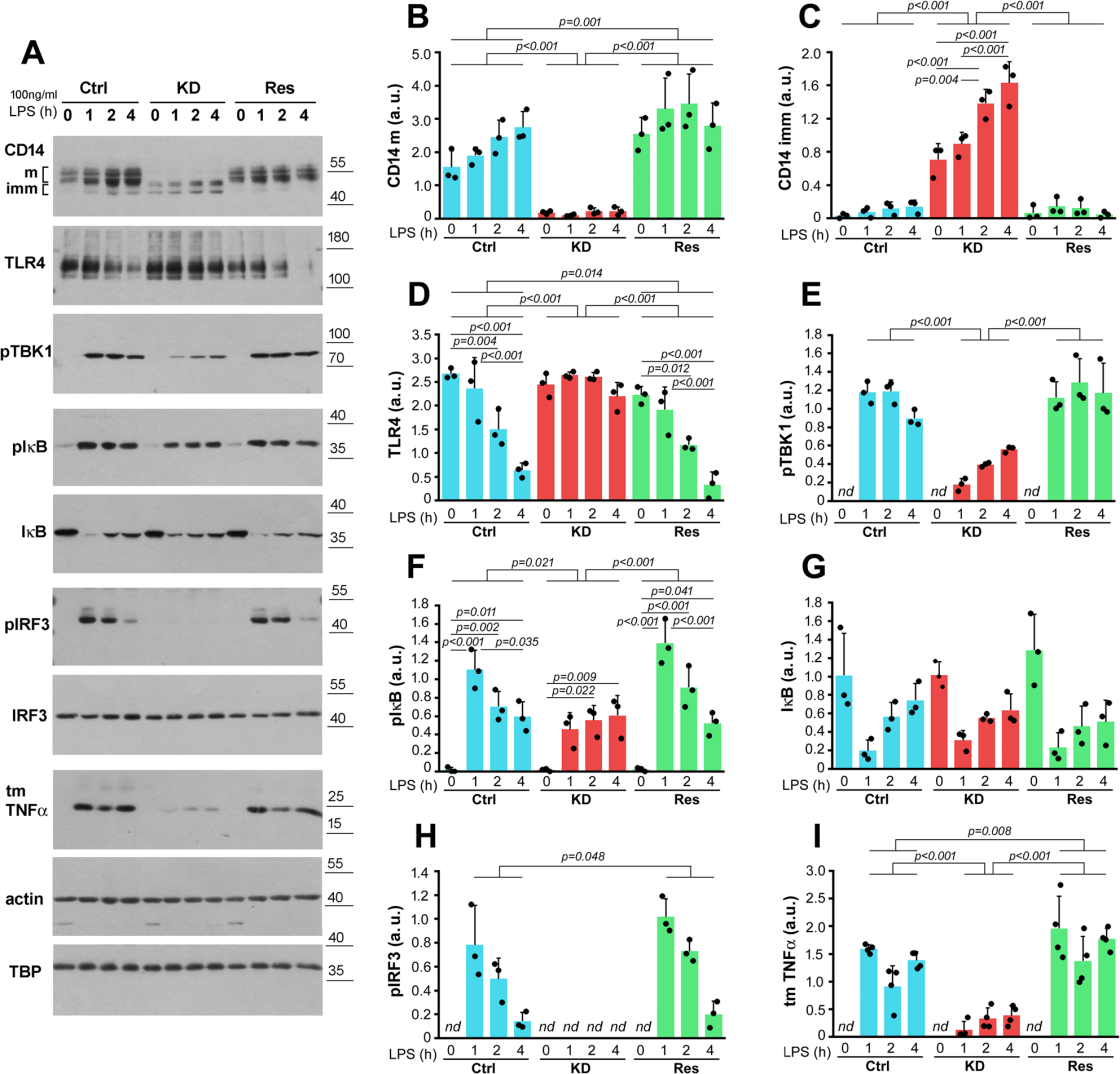
DGKε knockdown and rescue affect the abundance of mature CD14 and LPS-induced signaling. Cells were left unstimulated or were stimulated with 100 ng/ml LPS (1, 2 or 4 h, 37 °C). **A** Abundance of indicated proteins in the cells determined by immunoblotting. Positions of molecular weight markers are shown on the right in kDa. Actin and TBP were visualized to verify equal loading of protein between wells. **B**-**I** Abundance of mature CD14 (CD14 m) (**B**), immature CD14 (CD14 imm) (**C**), TLR4 (**D**), phosphorylated TBK1 (pTBK1) (**E**), phosphorylated IκB (pIκB) (**F**), IκB (**G**), phosphorylated IRF3 (pIRF3) (**H**), and transmembrane TNFα precursor (tm TNFα) (**I**) determined by densitometry and expressed relative to actin (**B**-**D**, **F**, **G**, **I**), TBP (**E**), and IRF3 (**H**). Data shown are mean ± SD from three (**B**-**H**) or four (**I**) biological replicates. Each point represents one biological replicate. Significantly different values, as indicated by two-way ANOVA with Tukey’s post hoc test, are marked. *nd*, not detected; excluded from statistical analysis

To gain deeper insight into the contribution of DGKε to TLR4-induced signaling, we monitored the abundance of relevant proteins over a time course of cell stimulation with LPS (100 ng/ml LPS, 1–4 h). Unexpectedly, we found a strong influence of DGKε on CD14 abundance; the mature forms of CD14 (bands estimated at 49–55 kDa) were nearly absent in DGKε-KD cells, both resting and stimulated with LPS for up to 4 h. Their slower gel migration is caused by CD14 glycosylation [27, 79]. Instead, an accumulation of a faster-migrating doublet of CD14 (bands estimated at 46 and 49 kDa), tentatively assumed to represent immature forms of CD14, was found in these cells (Fig. 5A-C). In DGKε-Myc-rescued cells, the mature CD14 forms were restored at a level exceeding that in Ctrl cells (Fig. 5A, B). This was in agreement with the higher relative DGKε mRNA level and enhanced DGKε-mediated SAG phosphorylation in these cells (see Figs. 1 and 2). The Ctrl cells responded to 4-hour LPS stimulation with a progressive accumulation of mature CD14 (Fig. 5A, B). In contrast to CD14, TLR4 gradually disappeared in LPS-stimulated Ctrl and DGKε-Myc-rescued cells, owing to its CD14-dependent internalization and degradation [13, 80]. Importantly, in the DGKε-KD cells lacking mature CD14, LPS stimulation did not induce the TLR4 disappearance (Fig. 5A, D).

Among the downstream components of TLR4 signaling, the activation (phosphorylation) of TBK1, a TRAF3 and TRAF6 effector [81–83], was significantly decreased in DGKε-KD cells relative to control throughout the 4-h period of LPS stimulation and fully restored in DGKε-Myc-rescued cells (Fig. 5E). The phosphorylation of IκB peaked at 1 h of LPS stimulation in both Ctrl and DGKε-Myc-rescued cells, and was lower by about 59–67% in DGKε-KD cells; its level was constant during the subsequent 3 h of stimulation in the DGKε-KD cells (Fig. 5F). IκB was degraded following its phosphorylation, with similar kinetics in all the cells (Fig. 5G). In contrast, the phosphorylation of IRF3 (activated strictly by endosomal TLR4) was nullified in the DGKε-KD cells but was restored in the DGKε-Myc-rescued cells at a level moderately increased relative to Ctrl cells (Fig. 5H). The depletion of DGKε and its restoration also substantially affected the accumulation of TNFα transmembrane precursor (Fig. 5I).

Taken together, these results strongly support a link between DGKε abundance and activity, and the level of mature CD14, which primarily determines the endosomal LPS-induced pro-inflammatory signaling of TLR4.

### DGKε affects the surface level of CD14 and other GPI-anchored proteins

**Fig. 6:**
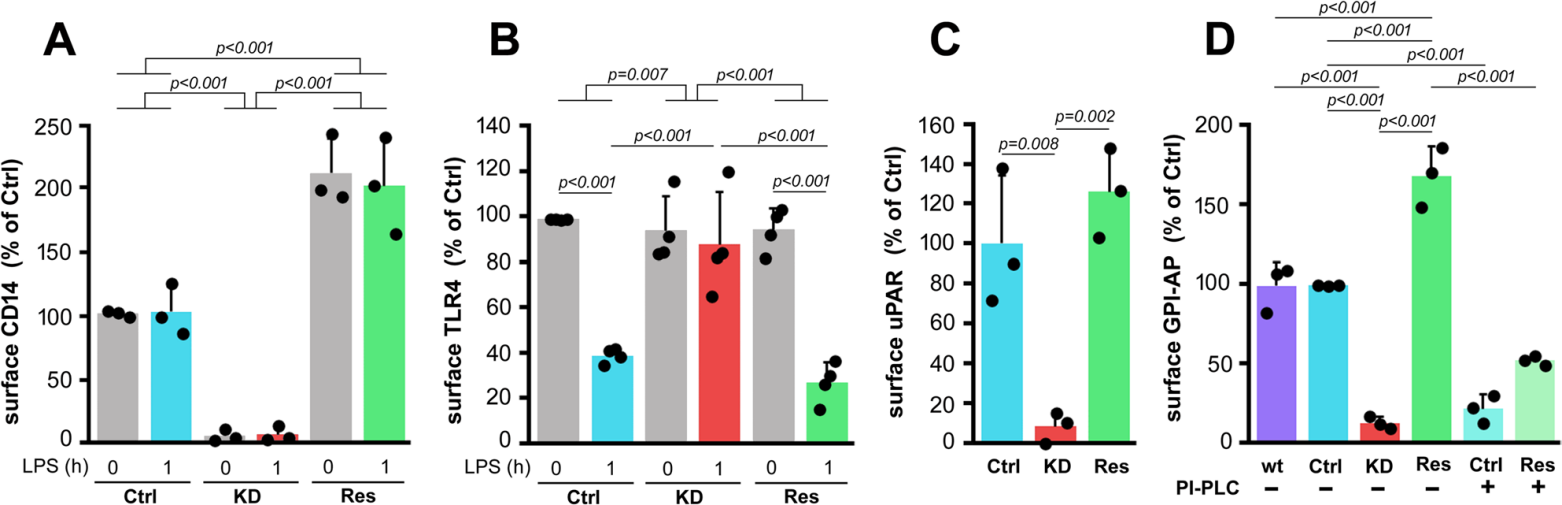
DGKε knockdown and rescue affect the cell-surface level of GPI-APs. Cells were left unstimulated or were stimulated with 100 ng/ml LPS (1 h). The cell-surface level of CD14 (**A**), TLR4 (**B**), uPAR (**C**), and total GPI-APs (**D**) was determined by flow cytometry. In (**D**), in a series of experiments unstimulated cells were pretreated with PI-PLC (0.2 U/ml, 1 h, 37 °C) before labeling with FLAER. Data are expressed as the percentage of the value in unstimulated Ctrl cells and are shown as mean ± SD from three (**A**, **C**, **D**) or four (**B**) biological replicates. Each point represents one biological replicate. Significantly different values, as indicated by two-way ANOVA (**A**, **B**) or one-way ANOVA (**C**, **D**), both with Tukey’s post hoc test, are marked

To further support the above conclusion, we analyzed the level of cell-surface CD14 by flow cytometry. Only residual cell-surface CD14 could be detected with an anti-CD14 antibody in DGKε-KD cells; CD14 reappeared on the surface of DGKε-Myc-rescued cells at a level ca. 2-fold higher than in Ctrl cells, both before and after a 1-h stimulation with LPS (Fig. 6A). In contrast to CD14, the surface level of TLR4 was comparable in all the cell types tested before LPS stimulation (Fig. 6B). The Ctrl and DGKε-Myc-rescued cells responded to LPS with a reduction of the cell-surface level of TLR4 by 61% and 72%, respectively, while no change was found in DGKε-KD cells (Fig. 6B). These results confirmed the absence of TLR4 endocytosis upon LPS treatment in the DGKε-KD cells and its restitution following the reintroduction of DGKε. Aside from CD14, the urokinase plasminogen activator receptor (uPAR), a GPI-AP found abundantly on the surface of Ctrl cells, was nearly absent in DGKε-KD cells and restored in DGKε-Myc-rescued cells (Fig. 6C). These findings were confirmed with the application of fluorescence-labeled inactive toxin aerolysin (FLAER), which binds selectively to the GPI anchor of a wide variety of GPI-linked proteins [84]. The FLAER binding was reduced by about 87% in DGKε-KD compared with wild-type and Ctrl cells and was restored in DGKε-Myc-rescued cells to a level surpassing the control 1.7-fold. Notably, the labeling with FLAER was strongly decreased in both Ctrl and DGKε-Myc-rescued cells pretreated with PI-PLC (by about 78% and 69%, respectively), indicating that the detected protein(s) were indeed GPI-anchored in the plasma membrane (Fig. 6D).

**Fig. 7:**
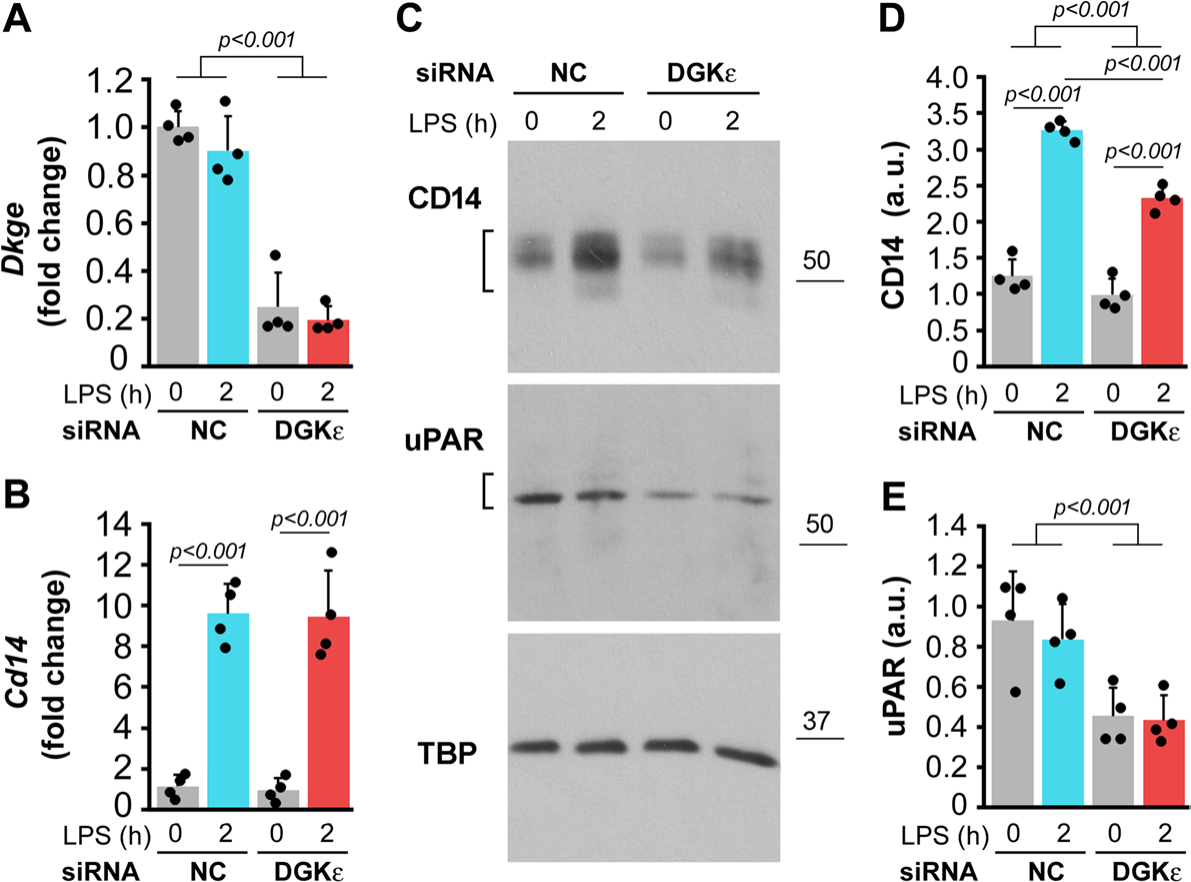
DGKε knockdown in BMDM reduces the abundance of CD14 and uPAR. BMDM were transfected with siRNA targeting DGKε or with negative control siRNA (NC) and left unstimulated or were stimulated with 100 ng/ml LPS (2 h). **A**, **B** *Dgke* and *Cd14* transcript was quantified by RT-qPCR relative to *Tbp* and *Hprt*, respectively, with unstimulated Ctrl set to 1. **C**-**E** Abundance of CD14 and uPAR was determined by immunoblotting and densitometry relative to TBP. Data shown are mean ± SD from four animals. Each point represents one animal. In (**A**, **D**, **E**), significantly different values, as indicated by two-way ANOVA with Tukey’s post hoc test, are marked. In (**B**), the two-way ANOVA indicated no significant differences between cell variants, therefore, differences between unstimulated and stimulated cells were analyzed with one-way ANOVA with Tukey’s post hoc test

Finally, we analyzed whether DGKε deficiency also affects the levels of GPI-APs in primary macrophages (BMDM). Transient silencing of *Dgke* in BMDM by about 75–78% (Fig. 7A) reduced the CD14 protein level in LPS-stimulated cells to about 30% (Fig. 7C, D). Concomitantly, the CD14 mRNA abundance remained unaffected by the *Dgke* silencing in both unstimulated and LPS-stimulated BMDM, with the increase observed in LPS-stimulated cells being pronounced (Fig. 7B). The silencing of *Dgke* in BMDM led to a significant reduction of uPAR protein up to 50% both in unstimulated and LPS-stimulated cells (Fig. 7C, E).

### DGKε determines the production of GPI-anchored CD14

**Fig. 8:**
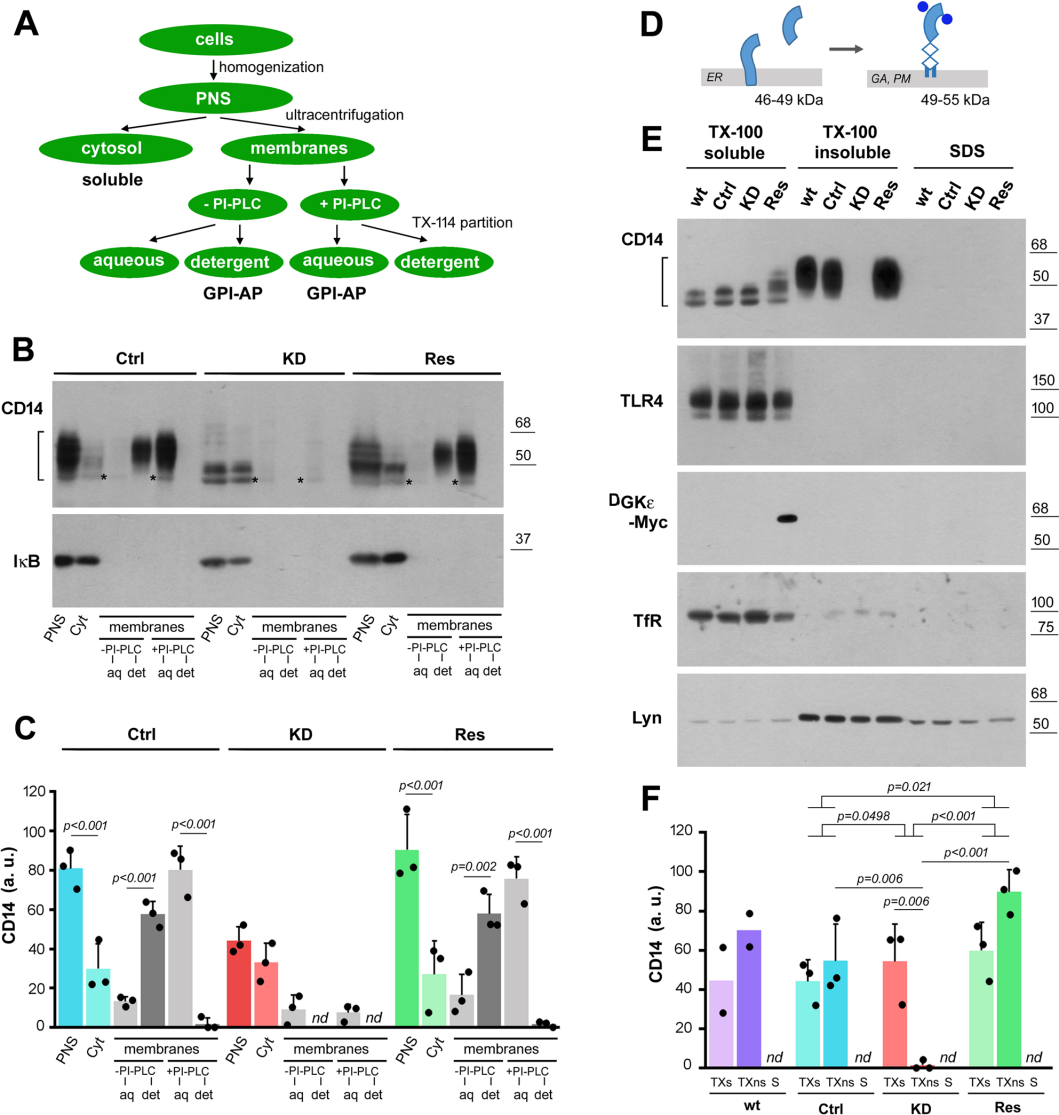
DGKε knockdown and rescue affect the abundance of GPI-anchored CD14. **A**-**C** Fractionation of cells with TX-114. **A** Scheme of the cell fractionation. Post-nuclear supernatants (PNS) obtained from homogenates of Ctrl, DGKε-KD, and DGKε-Myc-rescued cells were fractionated into cytosolic (Cyt) and membrane fractions. The membrane fraction was treated or not with 2 U/ml PI-PLC, lysed in 2% TX-114 and partitioned into aqueous (aq) and detergent (det) phases. **D** Schematic representation of CD14 forms. ER, endoplasmic reticulum; GA, Golgi apparatus; PM, plasma membrane. **E**-**F** Fractionation of cells with TX-100. Cells were solubilized in 0.1% TX-100 and fractionated into TX-100 soluble (TXs), TX-100 insoluble (TXns) and SDS-soluble (S) fractions. Equivalent volumes of the fractions were subjected to SDS-PAGE. **B**, **E** Distribution of indicated proteins in cell fractions determined by immunoblotting. Positions of molecular weight markers are shown on the right in kDa. In (**B**) asterisks mark a fast-migrating CD14 band in the aqueous phase of membranes. **C**, **F** Abundance of CD14 in cell fractions determined by densitometry. Data shown are mean ± SD from three biological replicates, except for wt in (**F**) performed in two biological replicates. Each point represents one biological replicate. Significantly different values, as indicated by one-way ANOVA (**C**) or two-way ANOVA (**F**), both with Tukey’s post hoc test, are marked. *nd*, not detected; excluded from statistical analysis

To get a further insight into the influence of DGKε on CD14 formation, we analyzed the distribution of CD14 in cytosolic and membrane fractions, the latter subjected to solubilization and fractionation with TX-114, taking advantage of the partition of GPI-APs to the detergent fraction [67] (scheme in Fig. 8A). The faster-migrating CD14 doublet (bands of 46 and 49 kDa), observed in all cell variants, was found in the cytosolic fraction, as marked by the presence of IκB (Fig. 8B, C). After solubilization and fractionation of membrane proteins with TX-114, the slow-migrating CD14 bands (49–55 kDa), present only in Ctrl and DGKε-Myc-rescued cells, were recovered in the detergent fraction, as could be expected for a GPI-AP [67]. They all shifted to the aqueous phase after PI-PLC treatment cleaving off the GPI moiety (Fig. 8B, C). The aqueous phase of all the cell types also contained small amounts of a fast-migrating CD14 form (estimated at 46 kDa) (Fig. 8B, asterisks). That would be a CD14 precursor containing the transmembrane moiety conferring its hydrophilic nature [85], as indicated in Fig. 8D.

Another characteristic feature of GPI-APs and other raft proteins is their insolubility in cold TX-100 [86]. After fractionation of cells into TX-100-soluble, TX-100-insoluble (also called DRM), and SDS-soluble (cytoskeletal) fractions, the mature forms of CD14 (49–55 kDa) accumulated in the DRM fraction of wild-type Raw264.7, Ctrl, and DGKε-Myc-rescued cells. Notably, in DGKε-KD cells, these CD14 forms were absent in the DRM fraction. All the faster-migrating forms of CD14 in all the cell variants were detected exclusively in the TX-100-soluble fraction (Fig. 8E, F), which also contained transferrin receptor (TfR) and TLR4, as expected [65], and DGKε-Myc in DGKε-Myc-rescued cells (Fig. 8E and Supplementary Fig. 4A-C). For a reason currently unknown, the TfR level was reduced significantly in DGKε-Myc-rescued cells (Fig. 8E and Supplementary Fig. 4C). Furthermore, the overall abundance and enrichment in the DRM fraction of Lyn kinase were similar in all the cell lines (Fig. 8E and Supplementary Fig. 4D). Taken together, considering the enzymatic activity of DGKε, the complete absence of GPI-anchored CD14 in DGKε-KD cells indicated a disturbed synthesis of the GPI moiety.

### Biosynthesis of GPI-anchored CD14 can be partially rescued by a synthetic GPI precursor

**Fig. 9:**
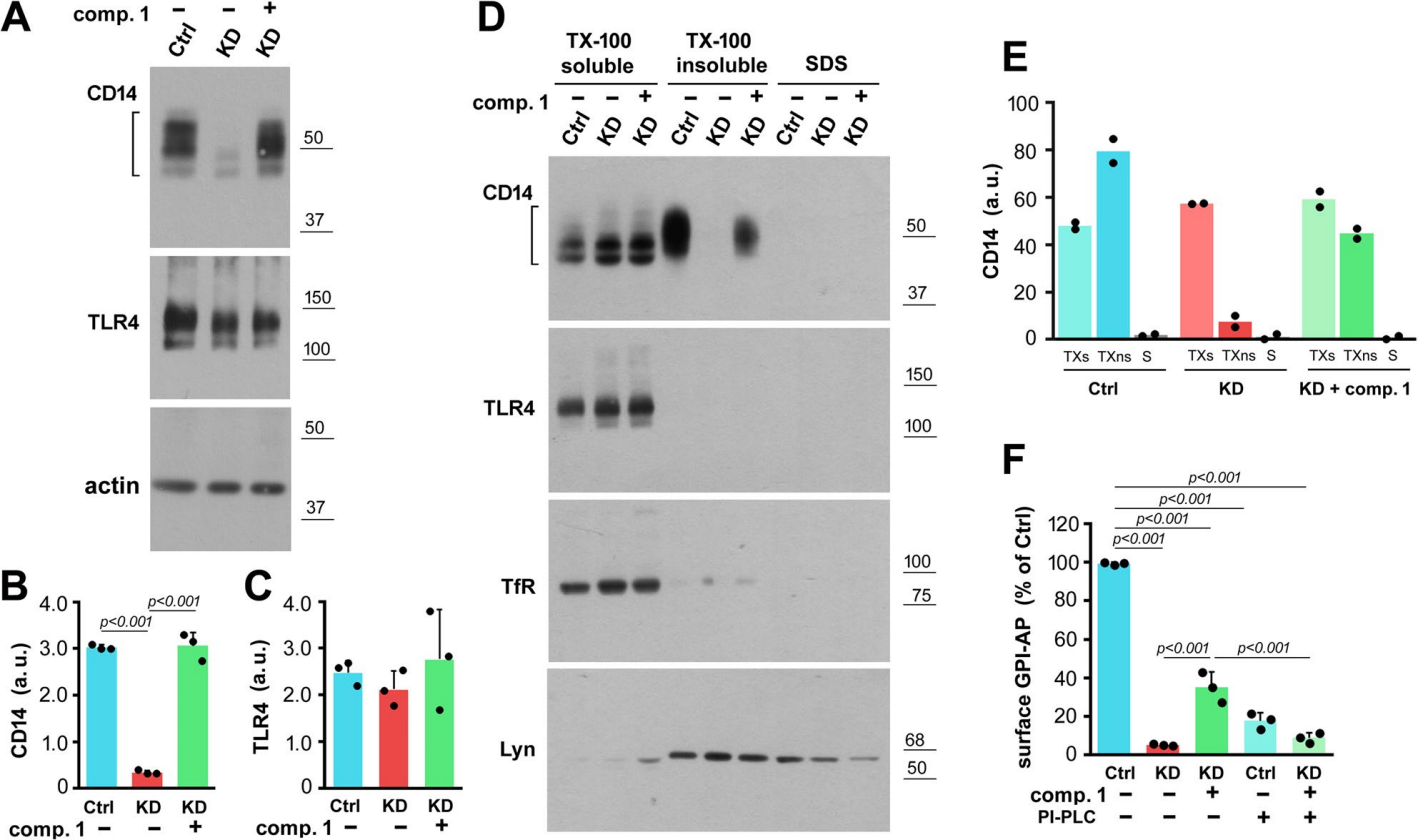
Synthesis of GPI-CD14 in DGKε-KD cells is rescued by compound 1. Cells were incubated with 50 µM compound 1 (+ comp. 1) or DMSO (− comp. 1). **A**-**C** Total cell lysates were analyzed for CD14 and TLR4 by immunoblotting (**A**) followed by densitometric analysis of their abundance relative to actin (**B**, **C**). **D**, **E **Fractionation of cells with TX-100. Cells were solubilized in 0.1% TX-100 and fractionated into TX-100 soluble (TXs), TX-100 insoluble (TXns) and SDS-soluble (S) fractions. Equivalent volumes of the fractions were subjected to SDS-PAGE and analyzed by immunoblotting (**D**) followed by densitometry (**E**) of CD14 abundance. **F** Flow cytometry analysis of total GPI-APs on the cell surface using FLAER staining. In a series of experiments, unstimulated cells were pretreated with PI-PLC (0.2 U/ml, 1 h, 37 °C) before labeling with FLAER. Data are expressed as the percentage of the value in Ctrl cells. In (**A**, **D**) positions of molecular weight markers are shown on the right in kDa. In (**B**, **C**, **F**) data are shown as mean ± SD from three biological replicates, in (**E**) the mean from two biological replicates. Each point represents one biological replicate. Significantly different values, as indicated by one-way ANOVA with Tukey’s post hoc test, are marked

To verify the involvement of DGKε in GPI anchor synthesis, we tested whether a supplementation of DGKε-KD cells with a synthetic GPI precursor could restore GPI-CD14 formation. For this purpose, DGKε-KD cells were treated with synthetic *N*-acetylglucosamine-phosphatidylinositol (GlcNAc-PI), the first PI derivative in the GPI biosynthesis pathway, referred to as compound 1 [64, 87]. Under these conditions, the CD14 synthesis was recovered, while TLR4 synthesis remained unaffected (Fig. 9A-C). The molecular weights of the newly synthesized CD14 forms were about 50–52 kDa, while forms of higher molecular weights (above 52 kDa) were less abundant (Fig. 9A). A substantial part of the CD14 species formed with compound 1-treated cells was insoluble in TX-100 (Fig. 9D, E), indicating their membrane-anchoring via GPI. The surface level of GPI-linked FLAER-stained proteins (including CD14) in these cells was increased compared to DGKε-KD cells, reaching about 35% of the control level; their membrane-association was lost following PI-PLC treatment (Fig. 9F). Taken together, the data underscore the key role of DGKε in the synthesis of GPI-linked CD14. However, they also suggest that DGKε may affect CD14 trafficking to the plasma membrane, and this activity cannot be fully substituted by compound 1.

### DGKε affects cytokine expression triggered by TLR2

**Fig. 10:**
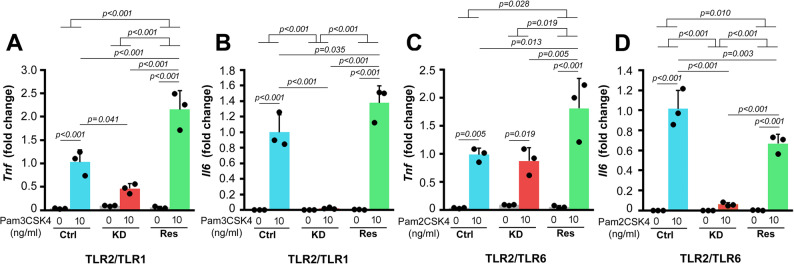
DGKε knockdown and rescue affect the expression of cytokines triggered by TLR2. Cells were left unstimulated or were stimulated with 10 ng/ml Pam_3_CSK_4_ (**A**, **B**) or 10 ng/ml Pam_2_CSK_4_ (**C**, **D**) (4 h). Transcripts of *Tnf* (**A**, **C**) and *Il6* (**B**, **D**) were quantified by RT-qPCR relative to *Hprt* and *Tbp*, respectively, with the stimulated Ctrl set to 1. Data shown are mean ± SD from three biological replicates. Each point represents one biological replicate. Significantly different values, as indicated by two-way ANOVA with Tukey’s post hoc test, are marked

To determine whether DGKε contributes to the pro-inflammatory signaling of TLRs other than TLR4, we treated the cells with Pam_3_CSK_4_ or Pam_2_CSK_4_ to activate TLR2/TLR1 or TLR2/TLR6 heterodimers, respectively. In the DGKε-KD cells, the induction of TNFα mRNA was reduced by about 55% following TLR2/TLR1 activation (Fig. 10A), whereas no significant difference was observed after TLR2/TLR6 activation (Fig. 10C). This is consistent with the stronger dependence of the TLR2/TLR1 complex on collaboration with CD14 for ligand binding [88]. In contrast, induction of *Il6* expression in response to either treatment was almost abolished in the DGKε-KD cells (Fig. 10B, D), suggesting that DGKε in macrophages affects TLR2 signaling beyond its effect on CD14. In DGKε-Myc-rescued cells, the response to either treatment was stronger (Fig. 10A-C) or only slightly weaker (Fig. 10D) than in the Ctrl cells.

## Discussion

CD14 is a GPI-anchored protein of the plasma membrane of myeloid cells, involved in TLR4 activation by facilitating LPS binding and governing endosomal signaling of TLR4 [14, 18, 22]. It also participates in the activation of several other TLRs [89]. In the present study, we show that DGKε is required for the cell-surface presentation of GPI-linked CD14. A stable depletion of DGKε in Raw264.7 macrophage-like cells (DGKε-KD) led to the disappearance of mature CD14 equipped with a GPI anchor destined for the cell surface. Moreover, transient silencing of *Dgke* in primary bone marrow-derived macrophages significantly reduced CD14 abundance. Since a reintroduction of DGKε in the DGKε-KD cells fully restored the GPI-CD14 formation, it is reasonable to conclude that the original defect was indeed due to the deficiency of DGKε and not to some unspecified effect caused by the *Dgke* silencing. The depletion and subsequent restoration of membrane GPI-linked CD14 resulted in an inhibition and recovery, respectively, of the CD14-dependent pro-inflammatory responses of TLR4 and TLR2.

The DGKε depletion/rescue did not affect the relative mRNA levels of CD14 or TLR4, indicating that DGKε affected CD14 at a post-transcriptional stage. Specifically, the lack of DGKε led to the disappearance of the mature, GPI-anchored forms of CD14 of a higher molecular weight caused by glycosylation [27] and localized on the cell surface [90]. In control and DGKε-Myc-rescued cells, these CD14 forms were enriched in the TX-100-insoluble fraction of the cell lysates. They also partitioned into the detergent-rich phase after solubilizing membrane proteins with TX-114, an effect that was abolished by PI-PLC treatment, as expected for GPI-APs. Taken together, these results indicate that the maturation of CD14 was impaired in DGKε-depleted cells, likely due to its defective modification with GPI. In support of this conclusion, the synthesis of GPI-CD14 (TX-100-insoluble) in DGKε-KD cells was substantially rescued by compound 1, GlcNAc-PI, a synthetic precursor of GPI. It has been established earlier that the attachment of the GPI moiety is indispensable for the exit of GPI-APs from the ER [25]. Therefore, the anterograde transport of newly synthesized CD14 was probably blocked in DGKε-KD cells. This was likely followed in part by its degradation [25] and in part by conversion to soluble CD14, as discussed below. Such a block would ultimately prevent the replenishment of the cell-surface pool of CD14, which is depleted by constitutive endocytosis and degradation occurring in resting macrophages [91].

**Fig. 11:**
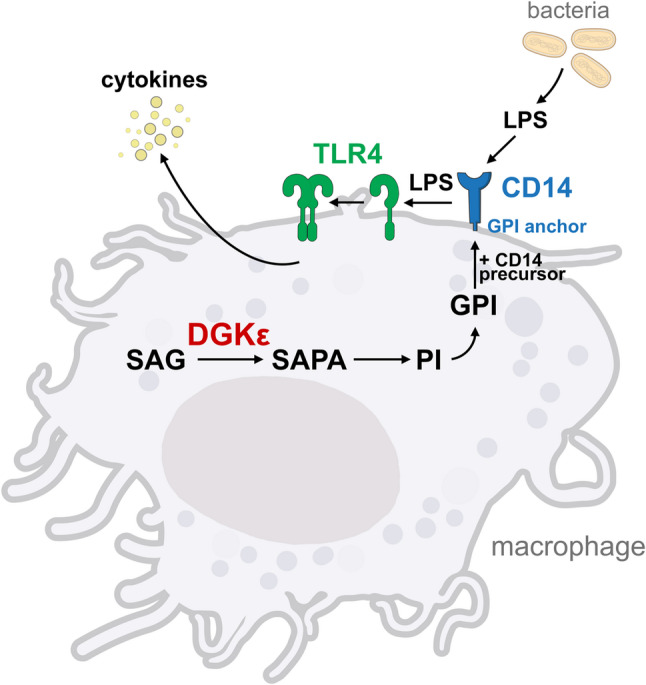
The SAG-to-SAPA phosphorylation by DGKε is required for PI synthesis, which in turn is essential for GPI-CD14 formation in macrophages, affecting pro-inflammatory signaling of TLR4

The biosynthesis of GPI is a multistep process initiated by the transfer of *N*-acetylglucosamine to the inositol ring of PI, forming GlcNAc-PI. The PI carries the C38:4 fatty acyl signature which is retained during the first two steps of the GPI anchor synthesis [46]. The initial step is catalyzed by a complex of phosphatidylinositol glycan anchor biosynthesis proteins (PIG proteins), with PIGA being the catalytic subunit. Both this step and the subsequent de-*N*-acetylation of GlcNAc-PI to GlcN-PI take place in the outer leaflet of the ER [25, 26], where the majority of DGKε is located [55]. The strict substrate specificity of DGKε predestines it to maintain the C38:4 fatty acid signature of PI [49]. On the other hand, Kim et al., [50] demonstrated that after transient silencing of *DGKE* in HEK293 cells, the resynthesis of C38:4 PI following agonist-induced PI(4,5)P_2_ hydrolysis can involve the participation of multiple DGK isoenzymes. Notably, in our hands, transient silencing of *Dgke* in BMDM reduced but did not abolish the formation of CD14 and uPAR. In contrast, a stable depletion of DGKε with shRNA in Raw264.7 cells, followed by DGKε restitution, revealed that the relative *Dgke* expression level was closely correlated with the efficiency of SAG phosphorylation in the membrane fraction stripped of other DGKs, and also with the abundance of GPI-linked CD14 and uPAR. These results indicate that the PI synthesis involving the DGKε-mediated SAG-to-SAPA phosphorylation is crucial for the synthesis of the PI pool used for the synthesis of the GPI anchor of CD14 (Fig. 11) and other GPI-APs in macrophages. Further lipidomic studies are required to unequivocally confirm this claim and to determine whether DGKε is involved in *de novo* PI synthesis and/or in the PI cycle proposed to operate under basal conditions, without agonist stimulation [61].

Recently, a sophisticated genetic screen using PIGA-KO HEK293 cells and compound 1 was performed to identify proteins involved in GPI biosynthesis. The CLPTM1L scramblase was found to aid the PIG-family proteins in GPI synthesis by mediating the translocation of GlcN-PI to the luminal leaflet of the ER for further steps of GPI synthesis [87]. Our results place DGKε activity upstream of PIGA and CLPTM1L in this pathway, potentially explaining why it could not be identified in the above study. On the other hand, it remains to be determined whether DGKε involvement in GPI-APs production in macrophages is also critical in other cell types. While DGKε is ubiquitously expressed, its expression level and expression patterns of the other nine DGKs are cell-type specific according to the Human Protein Atlas (proteinatlas.org). Nevertheless, comprehensive data on these expression patterns remain limited [92]. The cell-type-specific expression landscape of DGK isoenzymes can ultimately determine whether DGKε plays an exclusive or a redundant role in GPI formation. A cell-type-specific contribution of DGKε to GPI synthesis could explain why *PIGA* mutations in hematopoietic stem cells (and sporadically mutations in other *PIG* genes) lead to paroxysmal nocturnal hemoglobinuria (PNH), whereas inherited *DGKE* mutations are currently recognized as a cause of atypical hemolytic uremic syndrome (aHUS), a form of kidney disease. Interestingly, a recent study on *PIGA*-KO peripheral blood mononuclear cells from PNH patients found impaired TLR2-dependent activation of the NLRP3 inflammasome, which could be tentatively attributed to an absence of CD14 [93]. These findings are consistent with our results on reduced cytokine expression upon TLR2 activation in DGKε-KD cells and highlight certain similarities in the consequences of PIGA and DGKε deficiency in monocytes/macrophages following activation of TLRs.

The DGKε depletion and reintroduction affected most significantly the induction of the endosomal TLR4 signaling which requires CD14-mediated endocytosis of the receptor [14, 18] or/and the delivery of LPS by CD14 to intracellular TLR4 [22]. The magnitude of the MyD88-dependent TLR4 signaling was also affected, especially at the lower LPS concentration, reflecting the role of CD14 in delivering LPS to TLR4/MD2 [34, 35]. Furthermore, the MyD88-dependent expression of *Tnf* following TLR2/TLR1 activation was significantly inhibited by DGKε depletion, consistent with its sensitivity to antibody-mediated neutralization of CD14 [35]. Taken together, these data corroborate earlier findings that the pro-inflammatory signaling of TLR4 and TLR2 depends on the abundance of plasma-membrane CD14. The cell-surface level of CD14 in macrophages is determined by its complex cellular trafficking that differs substantially from that of TLR4 [69]. We have recently found that the TLR4 signaling was reduced by disturbances in CD14 trafficking. These include an inhibition of CD14 recycling, its up-regulation followed by enhanced shedding, and a depletion of sphingomyelin (a raft lipid), all ultimately causing a reduction of the total and the cell-surface abundance of CD14 [65, 70, 90]. Furthermore, oxPAPC-mediated endocytosis of CD14 and its clearance from the cell surface inhibited the subsequent LPS-induced responses [94]. In addition to the TLR signaling, CD14 participates in the non-canonical inflammasome activation by intracellular LPS [95]. The cell-surface CD14 also regulates the endocytosis and the pro-inflammatory activity of FAS receptor in macrophages and neutrophils [96]. These data suggest that the DGKε-dependent formation of GPI-linked CD14 likely affects several innate immune responses.

In Raw264.7 cells depleted of DGKε, only the cytosolic doublet of anchorless forms of CD14 was abundant. It resembled the doublet of truncated CD14 forms devoid of the C-terminal sequence, including the GPI attachment signal motif - the CD14-(1-335)-peptide described by Stelter et al., [27]. These truncated CD14 forms were released from cells, thereby serving as a source of soluble CD14 detected in the extracellular milieu. In view of our results, it seems likely that the anchorless CD14 forms are produced by proteolysis of the transmembrane precursor of CD14 in the ER. In the absence of DGKε and the consequent GPI depletion, these are the only forms of CD14 synthesized by the cells. In patients suffering from PNH, the serum level of soluble CD14 is comparable to that in healthy controls. Soluble CD14 supports at least some LPS-induced pro-inflammatory responses [97], pointing to the biological significance of this CD14 form.

Finally, our results indicate that the DGKε functions in macrophages are likely not limited to its role in GPI-APs formation. The evidence for this claim is the nearly complete inhibition of *Il6* expression following TLR2/TLR1 or TLR2/TLR6 activation in DGKε-KD cells, likely resulting from a decreased PI level. This suggestion is supported by the earlier observation showing that the PI3 kinase-mediated activation of AKT was required for IL-6 production, but not that of TNFα, in Kupffer cells upon TLR2/TLR6 activation [98]. Furthermore, compound 1 restored the cell-surface CD14 level to only about 35% of the control level, suggesting an impaired transport of CD14 to the plasma membrane. Also, TfR abundance was affected by DGKε, implying its involvement in TfR recycling. Altogether, these data suggest that DGKε can affect vesicular trafficking of proteins, potentially through its role in the synthesis of SAPA, PI, and other lipids. When undertaking our study, we assumed that the depletion/rescue of *Dgke* expression would allow us to demonstrate its involvement in the PI(4,5)P_2_ generation accompanying the LPS binding to CD14 [37, 43]. However, due to the unexpected effect of the DGKε depletion on CD14 maturation, this question could not be addressed using this simple approach.

As mentioned above, mutations of *DGKE*, some of which lead to DGKε degradation [63], cause aHUS [99, 100]. An involvement of DGKε in energy-lipid metabolism has been indicated by a series of studies conducted on DGKε-KO mice, in line with its localization in the ER. Thus, the DGKε-KO mice were prone to high-fat diet-induced obesity and insulin resistance due to alterations in triglyceride metabolizing enzymes in white adipocytes [101]; long-term high-fat diet feeding alleviated those symptoms following the remodeling of the adipose tissue in three different fat depots [102, 103]. In turn, cardiac-specific overexpression of DGKε protected mice from cardiac dysfunction induced by chronic pressure overload [104]. Our present results on TLR pro-inflammatory signaling expand the list of DGKε-dependent processes even further.

## Supplementary Information

Supplementary Material 1.

Supplementary Material 2.

## Data Availability

All data needed to evaluate the conclusions in the paper are present in the paper or the Supplementary Materials.
