# Supplementary material for: Diacylglycerol kinase-ε is required for the formation of GPI-anchored CD14 and the LPS-induced proinflammatory responses of macrophages
Source: Cell Commun Signal. 2026 Apr 25;24:344. doi: 10.1186/s12964-026-02884-2 (PMC13248311; doi:10.1186/s12964-026-02884-2)
Supplement: Supplementary file 1 — Supplementary Material 1. [file 12964_2026_2884_MOESM1_ESM.pdf]

Diacylglycerol kinase-ε is required for the formation of GPI-anchored CD14 and the LPS-induced proinflammatory responses of macrophages

Aneta Hromada-Judycka, Gabriela Traczyk, Ichrak Ben Amor, Anna Ciesielska, Aniela Mąkosa, Daniel Varon Silva, Katarzyna Kwiatkowska

Methods

Construction of Raw264.7 cells with silenced and rescued expression of *Dgke*

To obtain Raw264.7 cells depleted of DGKε, they were transfected with lentiviral particles (at MOI = 1 or 5) containing five different *Dgke*-targeting shRNA (Merck, Supplementary Table 1). Cells infected with transduction particles bearing non-mammalian shRNA (at MOI = 1 or 5) (Merck, Supplementary Table 1) served as controls. The cells were cultured in the presence of 2 μg/ml puromycin until the mortality of non-infected cells reached 100%, leaving only cells transfected with shRNA alive, essentially as described earlier (Traczyk *et al.,* 2022; Matveichuk *et al.,* 2024). To reintroduce DGKε, cells transfected at MOI = 5 with shRNA No. 1 and selected as described above were transfected (at MOI = 5) with lentiviral particles bearing the DGKε sequence with a double Myc tag added at the C-terminus, and conferring resistance to G418. The cDNA sequence was designed to contain eight point mutations in six codons, which did not change the amino acid sequence of DGKε but protected its mRNA from degradation guided by the previously introduced shRNA (Supplementary Table 1). Cells were cultured in the presence of 0.3 mg/ml G418 and selected as in the first round of transfection to obtain DGKε-Myc-rescued cells. In parallel, cells depleted of DGKε with shRNA No. 1 and also control cells obtained after the first round of transfection and selection (MOI = 5) were subjected to a second round of transfection with lentiviral particles bearing the G418 resistance gene only (at MOI = 5) and selected to obtain the ultimate DGKε knockdown cells (DGKε-KD) and control (Ctrl) cells. Lentiviral particles bearing the pLenti-C-Myc-DGGK-IRES-Neo vector, with or without the DGK-Myc coding sequence, were custom-made by OriGene. The efficiency of *Dgke* silencing and its reversion was verified with RT-qPCR using primers specific to the mouse *Dgke* gene, with *Tbp* as a reference (Supplementary Table 2, see also Traczyk *et al.*, 2022).

DGKε activity assay. Cell lysis and preparation of micelle

Raw264.7 cells (6 x 10^5 per sample) were lysed in 200 μl of lysis buffer (1% NP-40, 150 mM NaCl, 1 mM EDTA, 1 mM EGTA, 1 mM DTT, 1 mM PMSF, 20 μg/ml aprotinin, 20 μg/ml leupeptin, 20 mM Tris-HCl, pH 7.4) and sonicated (Traczyk *et al.,* 2022). Subsequently, 50 μl of the lysate containing 40 or 50 μg of total protein was used for the activity assay with 50 μl of micelle suspension, 100 μl of H_2O, and 0.5 mM ATP. The micelles were composed of NBD-SAG/SAG (at a 1:9 molar ratio, both from Cayman Chemical, cat. No. of SAG 10008650) and 1,2-dioleoyl-*sn*-glycero-phosphoserine (DOPS, Merck, cat. No. 840035C) at a final concentration of 1.45:2.03 mol% in 4 x reaction buffer containing 400 mM NaCl, 80 mM $MgCl_2$, 4 mM EGTA, 4 mM DTT, 300 mM octyl-β-glucoside, 200 mM MOPS, pH 7.2.

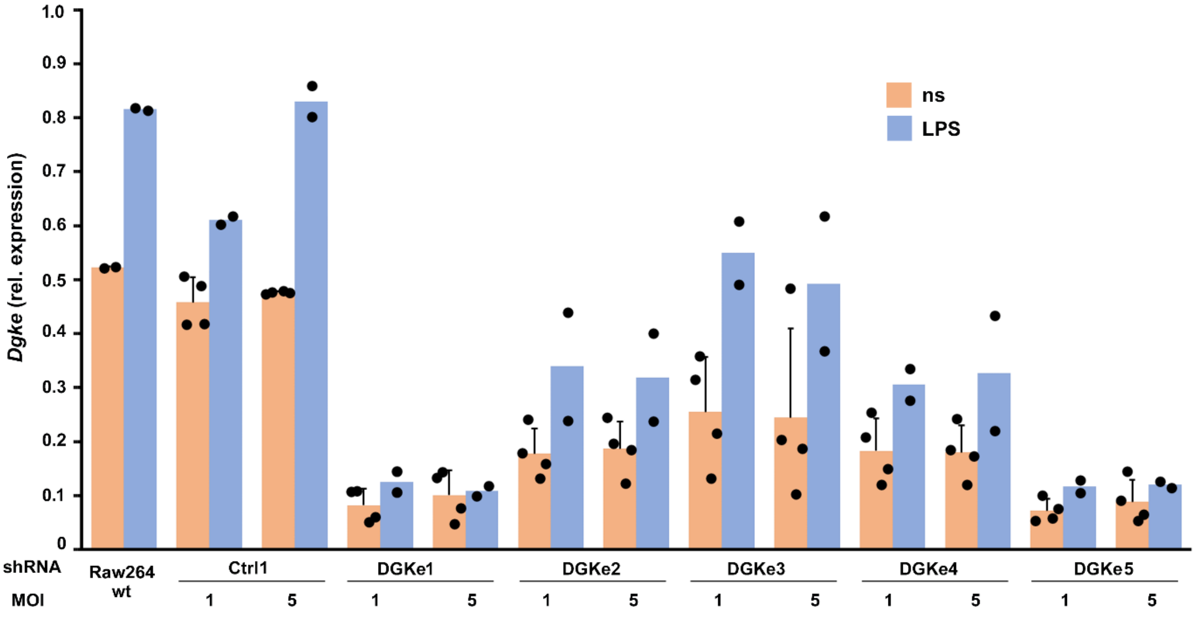

Supplementary Fig. 1. RT-qPCR analysis of the relative DGKε mRNA level in Raw264.7 cells after the knockdown of *Dgke* with shRNA. To silence *Dgke* expression, commercially available lentiviral particles bearing five shRNA variants were used individually (DGKe1-5) at MOI = 1 or 5, followed by puromycin selection. In parallel, control shRNA (Ctrl1) was applied at MOI = 1 or 5. Wild-type Raw264.7 cells – Raw264 wt. Cells were left unstimulated (ns) or were stimulated with 100 ng/ml LPS for 4 h. DGKε mRNA was quantified relative to TBP mRNA. Data shown are mean ± SD from four biological replicates of unstimulated cells, except for WT, which includes two biological replicates. For LPS-stimulated cells, the mean of two biological replicates is shown.

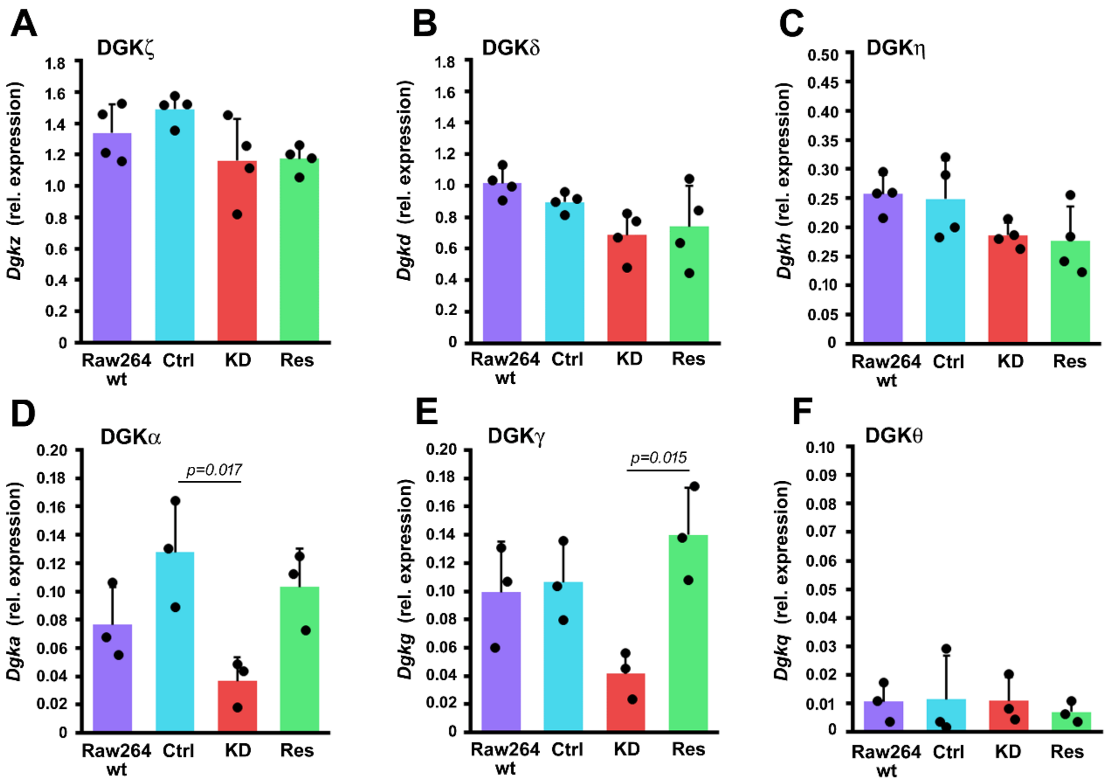

Supplementary Fig. 2. Relative expression levels of DGKs in Raw264.7 cells and their derivatives. Transcripts of *Dgkz* (A), *Dgkd* (B), *Dgkh* (C), *Dgka* (D), *Dgkg* (E), and *Dgkq* (F) were quantified relative to *Tbp*. Wild-type Raw264.7 cells – Raw264 wt. Data shown are mean ± SD from four (A-C) or three (D-F) biological replicates. Each point represents one biological replicate. Significantly different values, as indicated by one-way ANOVA with Tukey's post hoc test, are marked.

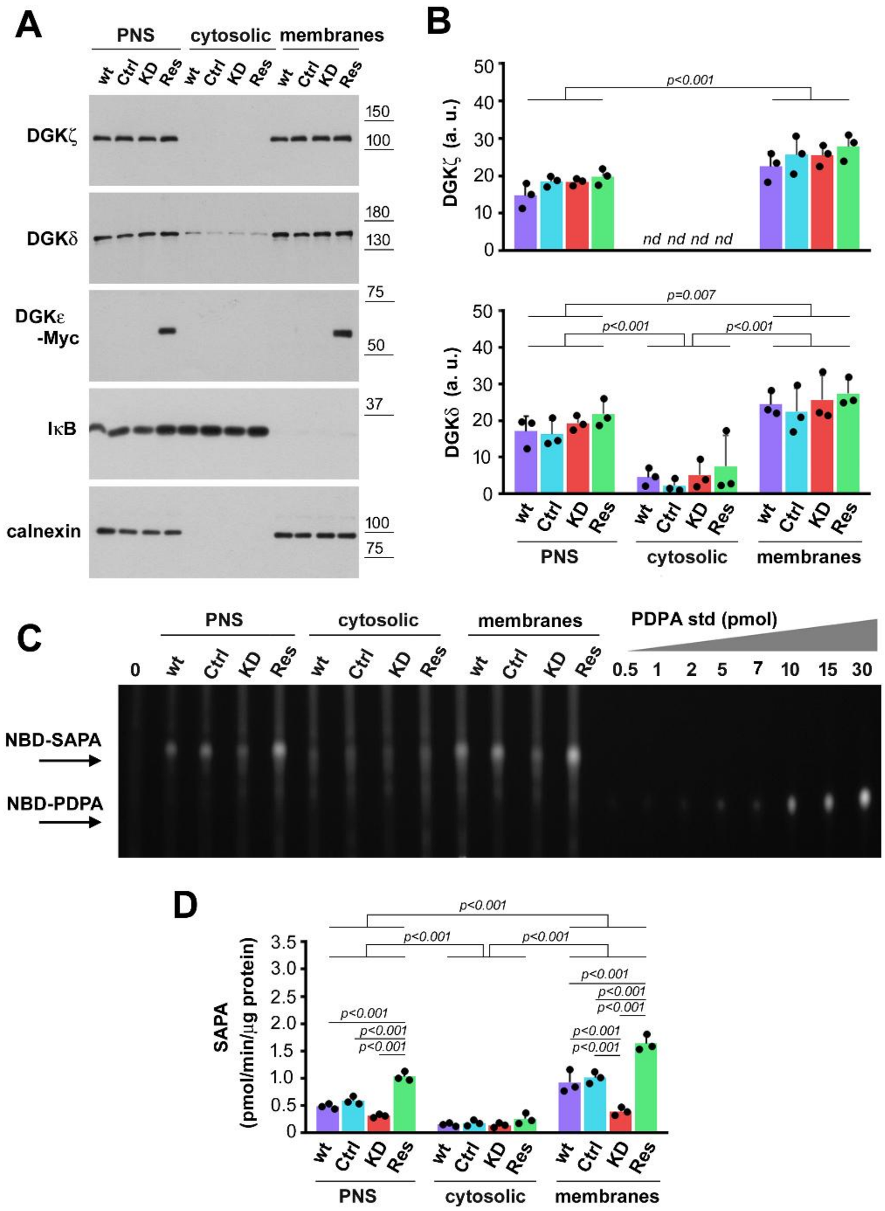

Supplementary Fig. 3. SAG phosphorylation in cell fractions not treated with 1 M NaCl. Post-nuclear supernatants (PNS) of wild-type Raw264.7 (wt), Ctrl, DGKε-KD, and DGKε-Myc-rescued cell homogenates were fractionated into cytosolic and membrane fractions without the addition of 1 M NaCl. (A) Distribution of indicated proteins in the fractions determined by immunoblotting. Equal amounts of protein, 10 µg, were loaded in each lane of the gel. Positions of molecular weight markers are shown on the right in kDa. (B) Abundance of DGKζ (upper panel) and DGKδ (lower panel) in each fraction determined by densitometry of blots such as those shown in (A). Data shown are mean ± SD. (C, D) Phosphorylation of SAG to SAPA in cell fractions determined using the fluorescence assay. (C) Representative TLC results revealing NBD-SAPA production. The reaction mixture contained 50 µg of total protein; lipids from 1/5 of the reaction mixture were applied onto the plate. NBD-labeled lipids were separated by TLC together with 0.5–30 pmol NBD-PDPA used to draw a standard curve for each experiment. “0” – no homogenate added. (D) SAPA production based on densitometric analysis of SAPA and a calibration curve for NBD-PDPA. Data shown are mean ± SD from three biological replicates. Each point represents one biological replicate. Because equal amounts of

protein were loaded on the gels and used for SAG phosphorylation, proteins in both the cytosolic and membrane fractions are enriched compared to the PNS. In (B, D) significantly different values, as indicated by two-way ANOVA with Tukey's post hoc test, are marked. *nd*, not detected; excluded from statistical analysis.

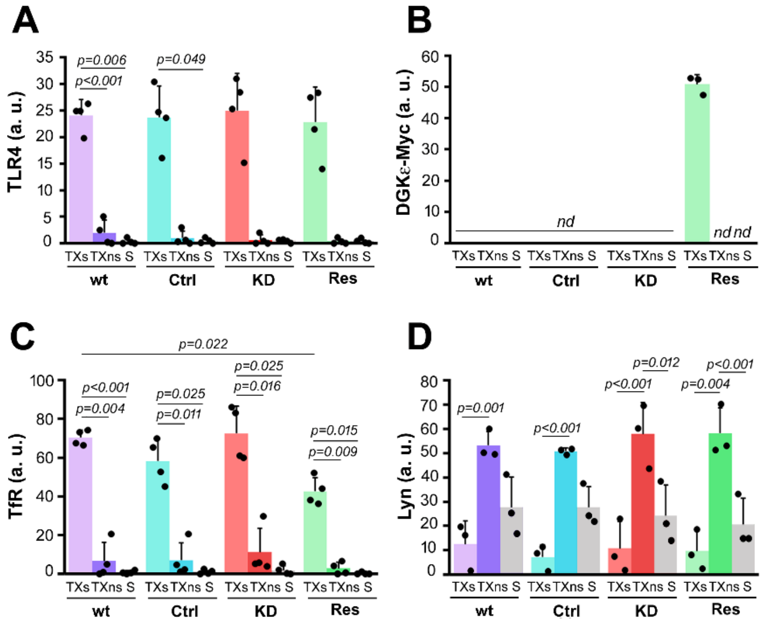

Supplementary Fig. 4. *Dgke* knockdown and rescue affect the abundance of mature forms of CD14. Cells were solubilized in 0.1% TX-100 and fractionated into TX-100 soluble (TXs), TX-100 insoluble (TXns), and SDS-soluble (S) fractions. Distribution of indicated proteins in the fractions determined by immunoblotting, as shown in Fig. 8 in the main text. (A-D) Abundance of TLR4 (A), DGKε-Myc (B), transferrin receptor (TfR) (C), and Lyn (D) in each fraction determined by densitometry. Data shown are mean ± SD from four (A, C) or three (B, D) biological replicates. Each point represents one biological replicate. In (A, C) significantly different values as indicated by Welch's ANOVA with Dunnett's T3 post hoc test are marked. In (D) the two-way ANOVA indicated no significant differences between cell variants, therefore differences between fractions in a given cell variant were analyzed with one-way ANOVA with Tukey's post hoc test. *nd*, not detected.

Supplementary Table 1. shRNA species used to silence *Dgke* and the *Dgke* sequence fragment with introduced silent point mutations to reverse *Dgke* silencing in rescue experiments

shRNA species used to silence *Dgke*			
Variant No.	Nucleotide sequence 5’- 3’	Merck catalog No.	Target sequence in the gene
1	CCGGCC**AAGCTACTTAACTTCTATT**CTCGAGAA<br>TAGAAGTTAAGTAGCTTGGTTTTT	TRCN0000024744	nt 921-939
2	CC**GGGCTGACTTTGAAGTGCTCCAT**CTCGAGAT<br>GGAGCACTTCAAAGTCAGCTTTTT	TRCN0000024745	nt 1877-1898
3	CCGGGC**CATTGATGAAATGAAGATT**CTCGAGAA<br>TCTTCATTTCATCAATGGCTTTTT	TRCN0000024748	nt 1221-1239
4	CCGG**CGCCATTTCACTCCTAGTATT**CTCGAGAA<br>TACTAGGAGTGAAATGGCGTTTTTG	TRCN0000274757	3’ UTR<br>nt 2119-2139
5	CCGGCC**AAGCTACTTAACTTCTATT**CTCGAGAA<br>TAGAAGTTAAGTAGCTTGGTTTTTG	TRCN0000274755	nt 921-939
control	CCGGCAACAAGATGAAGAGCACCAACTC GAGTT<br>GGTGCTCTTCATCTTGTTGTTTTT	SHC002V	----
Eight point mutations (pm) introduced into *Dgke* to reverse *Dgke* silencing			
Dgke WT<br>*Dgke*-8 pm	921 **AAGCTACTTAACTTCTATT** 939<br>921 **AAGTTATCTTACAAGTATC** 939<br>* * * * * * * * * * * *	self-designed, produced by OriGene	

Supplementary Table 2. List of primers used for RT-qPCR analysis of *Dgka, d, e, g, h, k, q* and *z* expression

**Target gene**	**Direction**	**Nucleotide sequence 5'- 3'**
Dgka	Forward	CGGACCTACCACAGCCTAAC
	Reverse	CCCACAGTCACACTCAGGAC
Dgkd	Forward	CCTCATTCACAGCATTCG
	Reverse	GCTACAGTTCAACCCCTC
Dgke	Forward	AGCCTAAACTGTGCGATTACA
	Reverse	TTGGCGGGATGATGAGGTTTC
Dgkg	Forward	CAGACTTCCGTGTTCTTGCC
	Reverse	CTTCGTAACCTCCTCCCCAG
Dgkh	Forward	AGGAAGTGTCACAGATGCGG
	Reverse	CGTGGGTTGGCTTTGTTCAG
Dgkk	Forward	AAGGGCTCAGAAACTCCAGC
	Reverse	CGTCGTCTCCGTCTCCCAGG
Dgkq	Forward	CTCCTGACTGCTGCCCTC
	Reverse	CCATCTCCACCACACACCA
Dgkz	Forward	CGAGAAGCCAACCCAGAGA
	Reverse	CCATTCCGTCACACACTACT

Supplementary Table 3. Antibodies used in the study

Specificity of antibody	Host	Supplier	Catalog No.	Dilution	Application
Primary antibodies					
actin	Mouse monoclonal IgG	BD Biosciences	#612656	1:15000	IB
CD14	Rat monoclonal IgG	BD Biosciences	#553738	1:4000-5000	IB
CD14	Rat monoclonal IgG2a-FITC	eBioscience	#11-0141-82	1:200	FC
F4/80	Rat IgG2a, κ-Alexa Fluor 647	BD Biosciences	#565854	1:300	FC
IκBα	Mouse monoclonal IgG	Cell Signaling Techn.	#4814	1:1000	IB
calnexin	Goat	Abcam	#ab192439	1:1000	IB
DGKζ	Rabbit	Abcam	#ab239081	1:1000	IB
DGKδ	Rabbit	Gift from Dr. Fumio Sakane, Ciba University*		1:1000	IB
IRF3	Rabbit monoclonal IgG	Cell Signaling Techn.	#4302	1:1000	IB
Lyn	Rabbit monoclonal IgG	Cell Signaling Techn.	#2732	1:2000	IB
Myc-tag	Mouse	Cell Signaling Techn.	#2276	1:1000	IB
phospho-IκBα	Rabbit monoclonal IgG	Cell Signaling Techn.	#2859	1:1000	IB
phospho-IRF3	Rabbit monoclonal IgG	Cell Signaling Techn.	#4947	1:1000	IB
phospho-TBK1/NAK	Rabbit monoclonal IgG	Cell Signaling Techn.	#5483	1:4000	IB
TLR4	Rabbit monoclonal IgG	Cell Signaling Techn.	#14358	1:1000	IB
TLR4	Rat monoclonal IgG2a-PE	BioLegend	#145404	1:200	FC
TNFα	Rabbit monoclonal IgG	Cell Signaling Techn.	#11948	1:1000	IB
transferrin receptor	Mouse	Thermo Fisher	#13-6800	1:2000-4000	IB
uPAR	Goat	Bio-Techne	#AF534	1:1000<br>1:300	IB<br>FC
Secondary antibodies/conjugated with					
goat IgG/HRP	Donkey	Rockland	#605-703-125	1:3000-1:5000	IB
goat IgG/ Alexa Fluor 647	Donkey	Thermo Fisher	#A21447	1:1200	FC
mouse IgG/ HRP	Goat	Jackson ImmunoResearch	# 115-035-003	1:6000-30000	IB
rabbit IgG/ HRP	Goat	Rockland	# 611-1302	1:8000	IB
rat IgG/HRP	Goat	Merck	# A9037	1:10000	IB
sheep IgG /HRP	Donkey	Jackson ImmunoResearch	# 713-035-003	1:6000	IB

IB, immunoblotting; FC, flow cytometry

*Sakane *et al.*, 1996, doi:10.1074/jbc.271.14.8394; Crotty *et al.*, 2006, doi: 10.1073/pnas.0604104103.
